# The transcriptome of the developing grain: a resource for understanding seed development and the molecular control of the functional and nutritional properties of wheat

**DOI:** 10.1186/s12864-017-4154-z

**Published:** 2017-10-11

**Authors:** Parimalan Rangan, Agnelo Furtado, Robert J. Henry

**Affiliations:** 1 Queensland Alliance for Agriculture and Food Innovation, University of Queensland, St Lucia, QLD 4072 Australia; 2 Division of Genomic Resources, ICAR-National Bureau of Plant Genetic Resources, New Delhi, 110012 India

**Keywords:** RNA-Seq, KEGG pathway mapping, Functional annotation, Wheat grains, Transcriptome, Global gene expression, Differential expression

## Abstract

**Background:**

Wheat is one of the three major cereals that have been domesticated to feed human populations. The composition of the wheat grain determines the functional properties of wheat including milling efficiency, bread making, and nutritional value. Transcriptome analysis of the developing wheat grain provides key insights into the molecular basis for grain development and quality.

**Results:**

The transcriptome of 35 genotypes was analysed by RNA-Seq at two development stages (14 and 30 days-post-anthesis, dpa) corresponding to the mid stage of development (stage Z75) and the almost mature seed (stage Z85). At 14dpa, most of the transcripts were associated with the synthesis of the major seed components including storage proteins and starch. At 30dpa, a diverse range of genes were expressed at low levels with a predominance of genes associated with seed defence and stress tolerance. RNA-Seq analysis of changes in expression between 14dpa and 30dpa stages revealed 26,477 transcripts that were significantly differentially expressed at a FDR corrected *p*-value cut-off at ≤0.01. Functional annotation and gene ontology mapping was performed and KEGG pathway mapping allowed grouping based upon biochemical linkages. This analysis demonstrated that photosynthesis associated with the pericarp was very active at 14dpa but had ceased by 30dpa. Recently reported genes for flour yield in milling and bread quality were found to influence wheat quality largely due to expression patterns at the earlier seed development stage.

**Conclusions:**

This study serves as a resource providing an overview of gene expression during wheat grain development at the early (14dpa) and late (30dpa) grain filling stages for use in studies of grain quality and nutritional value and in understanding seed biology.

**Electronic supplementary material:**

The online version of this article doi: (10.1186/s12864-017-4154-z) contains supplementary material, which is available to authorized users.

## Background

Human survival has relied heavily upon the food contained in the seeds of the three major cereals, rice, wheat and maize since the beginnings of agriculture [1]. Wheat (*Triticum aestivum* L.) feeds nearly 30% of the world population [2]. Bread wheat has a unique genetic makeup, being an allohexaploid (2n = 6× = 42) with three sub-genomes, A, B, D, and a novel seed composition due especially to the presence of the gluten proteins with characteristic features that enable the production of diverse food products [3]. The wheat caryopsis (dry seed) is comprised of three key tissues, the pericarp, embryo, and endosperm [4]. Anatomically, the largest part of the mature seed, the endosperm, consist mainly of starchy endosperm cells with an outer layer specialized layer known as the aleurone [5]. As the wheat grain matures, the viability of the starchy endosperm cells is lost while that of the aleurone cells is maintained [6]. The starchy endosperm cells contain 60-70% starch and 10-15% storage proteins and form the main food source in the seed [7].

The development of the wheat caryopsis begins following anthesis (flower development) and pollination and proceeds until a physiologically mature seed is formed. Based on morphological characteristics the process can be divided into distinct developmental stages [8] that have been classified, based upon the presence of distinguishable botanical structures, into five distinct stages, tissues undifferentiated (0-7 – days post anthesis (dpa)), embryo differentiation (7-14dpa), lateral root primordia initiation (14-21dpa), caps on lateral roots with appearance of primary leaves (21-31dpa), and fully differentiated (31-50dpa) [9]. Based on growth phases these five botanical stages have been placed into three distinct phases, grain enlargement (0-14dpa, Zadok’s scale: Z69-Z75), grain filling (15-35dpa, Z75-Z87), and physiological maturity (36-50dpa, Z87-Z92) [10, 11]. However this categorization may vary with genotype, growth environment, temperature, and other factors [12]. Structural and anatomical features of the maturing wheat caryopsis like the development of transfer cells [13], endosperm [4, 5, 7, 14–20] and aleurone [21] have been well studied. Accumulation of starch and storage protein in the starchy endosperm contributes 70–85% of the total grain yield [7] and has been intensely studied [4, 5, 15, 16, 18, 20, 22].

Although the anatomy and composition of the wheat caryopsis has been well studied there are few reports on analysis of the role of gene expression in controlling grain formation. There are studies of the transcriptome using microarray [23–25] and NGS based methods [26–28]; but each study investigated only a single cultivar. In a continuation of our earlier work identifying differentially expressed genes that underlie aleurone and starchy endosperm differentiation [29]; we now report a study of genes expressed during seed development across wheat cultivars.

**Fig. 1:**
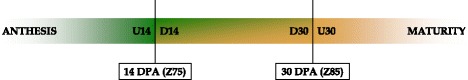
Schematic representation of developmental stages studied during wheat grain ontogeny. 14dpa: wheat grains sampled at 14 days-post-anthesis stage for transcriptome experiment; 30dpa: wheat grains sampled at 30 days-post-anthesis stage for transcriptome experiment; D14: differentially expressed transcripts at 14dpa; D30: differentially expressed transcripts at 30dpa; U14: uniquely expressed transcripts at 14dpa; U30: uniquely expressed transcripts at 30dpa

We report here, transcriptome profiling of the wheat caryopsis studied at two stages, at the boundary of grain enlargement and grain filing (14dpa, representing early grain filling) and at physiological maturity (30dpa, representing late grain filling) using RNA-Seq for 35 diverse cultivars (Fig. 1) [30]. The differences in expression and functional roles of these genes were characterized [31, 32] and their metabolic roles analysed [33]. In addition to explaining the role of gene expression in seed development, this study provides a platform for analysis of genetics of grain traits associated with grain yield, functional performance in food products and nutritional value in human and animal diets.

## Results

Sequence reads from 35 genotypes were mapped to the wheat cDNA database of *Triticum aestivum* gene indices (TaGI) containing 221,925 transcripts (TaGI, release 12.0). In total, 84.9% of the reads from 14dpa were mapped (Table 1 and Additional file 1); leading to an average 70× depth of sequencing. Reads from 30dpa provided a depth of 73× with 84.3% of reads mapped (Table 1 and Additional file 1).

**Table 1:** Summary statistics for RNA sequencing reads for 35 wheat genotypes at 14 and 30dpa^a^

	14dpa		30dpa	
	counts	%	counts	%
total reads	128,544,508		134,383,550	
paired-end reads	105,684,812	82.22	115,709,706	86.1
singleton reads	22,859,696	17.78	18,673,844	13.9
total mapped reads	109,067,249	84.85	113,234,886	84.26
mapped paired-end reads	89,905,504	82.43	97,660,733	86.25
mapped singleton reads	19,161,745	17.57	15,574,153	13.75

^a^For genotype specific details, please refer to Additional file 1

### RNA-Seq and differential gene expression analysis

The reads from 14dpa and 30dpa stages mapped against 178,767 and 174,695 transcripts respectively with 167,239 transcripts in common leaving 11,528 and 7456 mapped transcripts unique to the 14dpa and 30dpa stages. Of the 178,767 transcripts mapped at 14dpa stage, 116,148 sequences (~65%) were functionally annotated. Similarly, 112,608 transcripts were functionally annotated from 174,695 mapped transcripts at 30dpa stage. Transcripts from the 14dpa and 30dpa stages were functionally annotated and classified ontologically into three major aspects, biological process, molecular function, and cellular component using functional annotation software BLAST2GOv3.2.

### Functional annotation for mapped transcripts at 14dpa

The top five ontologies attributed to biological process at the 14dpa stage were; cellular component organization, cellular protein modification process, response to stress, catabolic process, and carbohydrate metabolic process. Similarly, for molecular function the top five categories were; nucleotide binding, DNA binding, kinase activity, transporter activity, and structural molecule activity. Cellular component was attributed to plastid, mitochondria, cytosol, ribosome, and plasma membrane. The complete set of biological process, molecular function, and cellular component classes were depicted in a cloud format with font size proportionately reflecting abundance at the 14dpa stage. Functional annotation analyses for the transcripts that are unique to the 14dpa stage (11,528 transcripts) revealed the top five biological process to be carbohydrate metabolic process, photosynthesis, cellular component organization, response to stress, and cellular protein modification process . In the case of molecular function, they were similar except for structural molecular activity being replaced with protein binding . Thylakoid cellular component replaces “ribosome” in the subset of transcripts that are unique to the 14dpa stage. The various biological process, molecular function and cellular component attributes that are unique to the 14dpa stage were organized in a cloud format with the font size reflecting their proportion .

### Functional annotation for mapped transcripts at 30dpa

Transcripts involved in biological processes like gluconeogenesis, response to misfolded protein, water transport, Golgi organization, and photosystem II assembly dominate during the 30dpa stage. Major molecular function ontologies mapped at this stage were ATP binding, zinc ion binding, structural constituent of ribosome, GTP binding, and heme binding. Key cellular components in which these biological process and molecular function ontologies makeup the biological system in a functional manner are plasmodesma, cytosolic large ribosomal subunit, apoplast, cytosolic small ribosomal subunit, and plastoglobule. The complete list of biological process ontologies at the 30dpa stage were depicted in a cloud format. Other minor molecular function and cellular component ontologies mapped during the 30dpa stage were also presented in cloud format. Mapped ontologies during the 30dpa stage for biological process, molecular function, and cellular component that constitute less than 1 % of the total expressed transcripts during the 30dpa stage were grouped in “others” category. Ontology mapping for the transcripts that are unique to the 30dpa stage (7456 transcripts) showed the domination of an entirely different set of biological processes, cellular oxidant detoxification, glyoxylate metabolic process, hydrogen peroxide catabolic process, sucrose metabolic process, and negative regulation of endopeptidase activity. Molecular function ontologies pertaining to the unique transcript sets at the 30dpa stage exhibited a similar pattern as at the 30dpa stage except “iron ion binding” which replaces “GTP binding”. Cytoplasmic, membrane-bound vesicle and transcription factor complex ontologies replace cytosolic small ribosomal subunit and plastoglobule ontologies among the top five cellular component ontologies when compared between the general set and the unique set of transcripts at 30dpa stage. A word cloud comprising all biological process, molecular function, and cellular component ontologies mapped with unique transcripts expressed at 30dpa stage was also generated.

### Differentially expressed transcripts between 14 and 30dpa

Differentially expressed transcripts between the 14dpa and 30dpa stages across all the genotypes were compared. False discovery rate (FDR) corrected *p*-values with 0.01 as cut-off were used to distinguish the differentially expressed transcripts that are statistically significant from the rest using statistical analyses (mean and count based) to harness the advantages from both the methods. In total, 34,737 and 34,378 transcripts (Table 2) were differentially expressed statistically for mean and count based methods respectively with a FDR cut-off at 0.01. A set of 26,477 transcripts (Table 2) were common to both statistical tests and are in four groups (Fig. 1) based on fold changes, unique transcripts at 14dpa (U14, 319); higher expression at 14dpa (D14, 16,237 – conversely downregulated at 30dpa); higher expression at 30dpa (D30, 9740 – conversely downregulated at 14dpa); and unique transcripts at 30dpa (U30, 181). Please refer to Additional files 2, 3, 4 and 5 for transcript details, fold changes, and statistics across all genotypes; and Additional files 6, 7, 8 and 9 for the sequence details of the respective transcript IDs. These four groups of transcripts were subjected to functional annotation independently using BLAST2GO [31, 32]. Of the U14 (319), D14 (16237), D30 (9740), and U30 (181) group of transcripts subjected to mapping and annotation, 191 (U14); 12,792 (D14); 6513 (D30); and 75 (U30) transcripts (Table 2) were annotated with one or more gene ontology terms and functions (Additional files 10, 11, 12 and 13). Pathway mapping using KEGG [33, 34] from BLAST2GO [31, 32] and filtering to discard non-plant related pathways yielded 52 (U14); 2791 (D14); 1121 (D30); and 11 (U30) transcript IDs (Table 2) that were mapped to various metabolic pathways (Table 3). Additional file 14 provides a list of pathways, differentially expressed transcript IDs linked with annotated enzymes involved in metabolic pathways. There were 70 and 71 metabolic pathways (Additional files 15, 16, 17 and 18) mapped from the transcriptome at 14 and 30dpa respectively through KEGG pathway mapping of BLAST2GO of which 68 were common. Key genes involved in different areas of metabolism that are significantly differentially expressed between 14dpa and 30dpa stages were tabulated (Table 4) and the most prominent ones are discussed below. Further details on the significantly differentially expressed genes between early and late grain filling for each of the nine areas of metabolism were documented as additional information (Additional file 19).

**Table 2:** Summary statistics on functional annotation and metabolic pathway mapping for differentially expressed transcript IDs

Details		U14	D14	D30	U30	Total
Differentially expressed transcripts @ FDR 0.01 cut-off	EDGE statistics	451	17,319	16,252	356	34,378
	GAUSS statistics	483	24,050	9980	224	34,737
	Common to both EDGE and GAUSS	319	16,237	9740	181	26,477
Mapped and annotated		191	12,792	6513	75	19,571
Transcript IDs mapped to various metabolic pathways after curation		52	2791	1121	11	3975

U14: transcripts expressed uniquely at 14dpa (days-post-anthesis); D14: transcripts differentially upregulated at 14dpa; D30: transcripts differentially upregulated at 30dpa; U30: transcripts expressed uniquely at 30dpa; EDGE: count based statistics; GAUSS: mean based statistics

**Table 3:** Summary of pathways across developmental stages grouped based on metabolic activities^a^

Metabolic activities	U14	D14	D30	U30
Nucleotide metabolism	2	3	3	1
Amino acid metabolism	3	19	22	6
Carbohydrate metabolism	4	7	7	3
Respiratory pathways	1	7	7	1
Photosynthesis	2	3	2	1
Lipid metabolism	2	13	13	–
Hormone biosynthesis	–	3	2	1
Vitamin metabolism	1	7	7	1
Specialized metabolism	1	8	8	1

^a^For list of various metabolic pathways that are grouped under various metabolic activities, please refer Additional files 14

**Table 4:** List of key genes differentially regulated between the 14dpa and 30dpa stages

S. No	Metabolism involved	Pathway involved	Key regulatory gene differentially expressed	Stages expressed
1	Nucleotide metabolism	Aminoacyl-tRNA biosynthesis	Ligase	D14 and D30
2	Nucleotide metabolism	Aminoacyl-tRNA biosynthesis	Synthase (Glutamine-hydrolysing)	D30
3	Amino acid metabolism	Phenylalanine metabolism	Lactoperoxidase	U14, D14, D30 and U30
4	Amino acid metabolism	Histidine; Lysine; Arginine and proline; cysteine and methionine; alanine, aspartate and glutamate; phenylalanine, tyrosine and tryptophan metabolism	Transaminase	D14
5	(Photorespiration; T-subunit of GDS)	Glycine, Serine and Threonine metabolism	S-aminomethyl dihydro lipoylprotein:(6S)-tetrahydrofolate aminomethyltransferase (ammonia-forming)	D14 and D30
6	(Photorespiration; P-subunit of GDS)	Glycine, Serine and Threonine metabolism	dehydrogenase (aminomethyl-transferring)	D14
7	Carbohydrate metabolism	Fructose and mannose; and galactose metabolism	Phosphohexokinase	D14 and D30
8	Carbohydrate metabolism	Amino sugar and nucleotide sugar; fructose and mannose; galactose; and starch and sucrose metabolism	Hexokinase type IV glucokinase	D14 and D30
9	(Photorespiration)	Glyoxylate and dicarboxylate metabolism (under carbohydrate metabolism)	Transaminase	D14
10	Respiratory pathways	Pentose phosphate pathway; Glycolysis / gluconeogenesis metabolism	Phosphohexokinase	D14 and D30
11	Respiratory pathways	Glycolysis / gluconeogenesis metabolism	Hexokinase type IV glucokinase	D14 and D30
12	Photosynthesis	Porphyrin and chlorophyll metabolism	protoporphyrin IX methyltransferase	D14
13	Photosynthesis	Porphyrin and chlorophyll metabolism	Porphobilinogen synthase	D14
14	Photosynthesis	Porphyrin and chlorophyll metabolism	Protoporphyrinogen oxidase	D14
15	Photosynthesis	Carbon fixation in photosynthetic organisms	Carboxylase (RuBisCO)	U14, D14, and D30
16	Photosynthesis	Carbon fixation in photosynthetic organisms	Carboxylase (phosphoenolpyruvate)	D14 and D30
17	Photosynthesis	Carbon fixation in photosynthetic organisms	Transaminase (Aspartate; EC2..6.1.1)	D14
18	Photosynthesis	Carbon fixation in photosynthetic organisms	Dehydrogenase (malate)	D14 and D30
19	Photosynthesis	Carbon fixation in photosynthetic organisms	Dehydrogenase (decarboxylating)	D14 and D30
20	Photosynthesis	Carbon fixation in photosynthetic organisms	Transaminase (alanine; EC2.6.1.2)	D14 and D30
21	Photosynthesis	Carbon fixation in photosynthetic organisms	Phosphate dikinase	D14
22	Lipid metabolism	Fatty acid biosynthesis; fatty acid degradation	Ligase (EC6.2.1.3)	D14
23	Lipid metabolism	Fatty acid biosynthesis	Hydrolase (EC3.1.2.14)	D14
24	Lipid metabolism	Fatty acid elongation	Hydrolase (EC3.1.2.22)	D14
25	Lipid metabolism	Glycerophospholipid metabolism; ether lipid metabolism; arachidonic acid metabolism; linoleic acid metabolism; alpha-linolenic acid metabolism	Phospholipase A2	D14 and D30
26	Hormone biosynthesis	Steroid biosynthesis	Monooxygenase	D14
27	Vitamin metabolism	One carbon pool by folate	Reductase	D30
28	Specialized metabolism	Phenylpropanoid metabolism	Ammonia-lyase	D14 and D30
29	Specialized metabolism	Terpenoid backbone biosynthesis	Geranyl-diphosphate synthase	D14 and D30

U14: transcripts expressed uniquely at 14dpa (days-post-anthesis); D14: transcripts differentially upregulated at 14dpa; D30: transcripts differentially upregulated at 30dpa; U30: transcripts expressed uniquely at 30dpa

## Discussion

Early analysis of differentially expressed genes in this data set facilitated the discovery of a gene associated with bread making quality (*wbm*) of wheat [35] that led us to study the global gene expression pattern across all genotypes of this study between the 14dpa and 30dpa stages. More recently, pathway mapping of this data through KEGG helped us discover a C_4_ pathway of photosynthesis without Kranz anatomy in wheat grains [36]. A compilation of the differentially regulated genes between 14dpa and 30dpa stages is now presented in this article (Additional file 14; Table 4). Based on the metabolism in which the pathways were involved, the 73 pathways were grouped into nine broad categories, nucleotide metabolism; amino acid metabolism; carbohydrate metabolism; respiratory pathways; photosynthesis; lipid metabolism; hormone biosynthesis; vitamin metabolism; and specialized metabolism (Table 3; Additional file 14). Although some metabolic pathways are highly conserved across taxa [37] like carbohydrate metabolism, respiratory pathways and nucleotide metabolism, others are highly diversified and specialized in some taxa [38].

Carbohydrate metabolic processes and photosynthesis are the dominant biological process ontologies identified at the 14dpa stage suggesting the likely importance of ear photosynthesis in determining grain yield [39, 40]. Phosphohexokinase and hexokinase type IV glucokinase are the key regulatory genes involved in carbohydrate metabolism differing between early and late grain filling stages. Transaminase that converts serine to hydroxy-pyruvate leading to glycerate formation (glyoxylate and dicarboxylate metabolism) is the key differentially expressed gene during early-grain filling that is linked with the photorespiration process (Table 4; Additional files 14 and 19). This data from carbohydrate metabolism also correlates well with photosynthesis and photorespiration being tightly linked and differentially regulated during the early-grain filling stage (14dpa). Key genes that are expressed differentially in carbohydrate metabolism, phosphohexokinase and hexokinase type IV glucokinase are also involved in respiratory metabolism (Additional file 14).

Photosynthesis is one of the crucial metabolic process that directly impacts on yield of the wheat grain. Genes involving porphyrin and chlorophyll metabolism are differentially expressed during early grain filling stage (Table 4). Genes involving C_4_ photosynthesis are differentially expressed between early and late grain filling stages leading us to discover the C_4_ photosynthetic pathway in the pericarp tissues of wheat grain [36]. The anatomy with which non-Kranz C_4_ photosynthetic pathway being accomplished were termed as Bose anatomy [41] in honour of his discovery on the role of malic acid (4C) in photosynthesis in *Hydrilla* sp. that was much later known to be single-cell C4 type. The RuBisCO small subunit was also differentially expressed during different developmental stages (Table 4; Additional file 14).

Amino-acyl tRNA biosynthesis with its ligases and synthase (glutamine-hydrolysing) are the key differentially expressed genes between early and late grain filling in nucleotide metabolism (Table 4 and Additional file 19). Transaminase is the key gene being differentially regulated for various types of amino acid metabolism (Table 4 and Additional file 14). It is also the best example of a transcripts that is being differentially expressed in different metabolic pathways of amino acid metabolism (Additional files 14 and 19). Lactoperoxidase of phenylalanine metabolism under amino acid metabolism is the best example for the transcript IDs that are differentially expressed across the early and late grain filling stages (Additional files 14 and 19). Tetrahydrofolate (THF) dependent aminomethyltransferase (EC2.1.2.10) of glycine, serine and threonine metabolism is a key differentially expressed gene in D14 and D30 stages (Table 4) and involved in photorespiration process of glycine decarboxylase system (GDS) by being the T-subunit component [42]. Similarly, P-subunit of GDS, dehydrogenase (aminomethyl-transferring; EC1.4.4.2) from glycine, serine and threonine metabolism is the key differentially expressed gene only during the D14 stage and is involved in the photorespiration process (Table 4).

Phospholipase A2 is the key differentially expressed gene that is involved in multiple pathways of lipid metabolism, glycerophospholipid metabolism, ether lipid metabolism, arachidonic acid metabolism, linoleic acid metabolism, and alpha-linolenic acid metabolism (Table 4). Except for ether lipid metabolism which is differentially expressed only during 14dpa stage, the others are differentially expressed both at 14dpa and 30dpa stages with different transcript IDs during different developmental stages (Additional file 14). Different hydrolase genes that are pathway specific and involved in fatty acid biosynthesis and fatty acid elongation pathways are differentially expressed at 14dpa stage; while ligase genes are differentially expressed at 14dpa stage and involved in fatty acid biosynthesis and degradation pathways (Table 4).

The monooxygenase gene is the key differentially expressed transcript at 14dpa stage involved in the steroid biosynthetic pathway and categorized under hormone biosynthesis (Table 4; Additional file 14). There are other monooxygenase genes that are differentially expressed and involved in amino acid metabolism, lipid metabolism and specialized metabolism and hence caution is required while manipulating this gene. Reductase is the key differentially expressed gene and rate limiting step during late-grain filling for one carbon pool by folate pathway being categorized under Vitamin metabolism (Table 4; Additional file 14). It is involved in conversion of folate to dihydrofolate and tetrahydrofolate (THF) – involved in photorespiration – involved in glycine, serine and threonine metabolism. This suggests that silencing of reductase (EC1.5.1.3) in plants might downregulate THF formation and in turn possibly minimize photorespiration. However, THF is known to be involved as a co-factor in various other one carbon metabolic processes like purine biosynthesis, pantothenate biosynthesis, organellar protein biosynthesis, and methionine biosynthesis [43]. So, how far the downregulation of reductase could be used in selectively restricting photorespiration during the late-grain filling stages without affecting the normal physiology of the plant is unclear. Ammonia-lyase and geranyl-diphosphate synthase respectively for phenylpropanoid and terpenoid backbone metabolic pathways involved in specialized metabolism are key genes that are differentially expressed at the 14dpa and 30dpa stages (Table 4; Additional files 14 and 19).

## Conclusion

This study provides an atlas of transcripts expressed during early (14dpa) and late (30dpa) grain filling in wheat. Among those, the key transcripts involving various metabolic pathways that are significantly differentially expressed at grain filling stages during wheat grain development were compiled (Additional file 14). Next generation sequencing (NGS) based transcriptome analysis (RNA-Seq) in combination with functional annotation has proved to be a very robust tool helpful in discovering novel genes such as the wheat bread making gene [35] and genes controlling flour yield and entire metabolic pathways (grain specific C_4_ photosynthesis) [36], improving our understanding on the complex metabolic networks at different developmental stages. The present resource will facilitate breeding selection for key genes in a metabolic pathway or a key metabolic pathway in a network involving specific developmental stages. Selection of genes expressed during grain filling identified in this dataset might be used for improving yield and grain quality. For example, a focus on silencing the genes that are involved in photorespiration, H-subunit (protein component with no independent catalytic activity) or P-subunit (Aminomethyl-transferring dehydrogenase) or T-subunit (THF dependent aminomethyltransferase) might be helpful in improving grain yield through reduced photorespiration [44]. Reducing photorespiration might be a significant contribution to achieve the goal of 20:20 Wheat® [45] - to achieve the productivity of 20 t per hectare in 20 years - through increased photosynthetic efficiency.

## Methods

### Plant material

The following 35 genotypes representing different geographical regions across the world were used for this study: Amurskaja, Arnhem, Banks, Bativa, Beyrouth-3, Bobwihte-26, Bowerbird, Des-367, Dollarbird, Ellison, Garbo, Giza-139, Gregory, Huandoy, India-37, India-211, India-259, Iraq-46, JingHong-1, Kite, LermaRojo, Martonvasari-13 T, Punjab-7, Qalbis, Saturno, Sunco, Sphaerococcum, Tunis-24, Greece-25, NW-25A, NW-51A, NW-93A, NW-108A, Pelada, and Vega. Seeds for these genotypes were obtained from the Australian Winter Cereals Genetic Resource collection and grown under controlled conditions (12 h light; 20 °C/18 °C day and night temperature; 70% Relative Humidity) in a growth cabinet as reported earlier [35].

### Sample collection and RNA extraction

Tagging, sample collection and pulverization using tissuelyzer (Qiagen, US) were done as described by Furtado et al. (2015) [35]. Wheat spikes were collected at 14 and 30 days-post-anthesis (dpa) stage (Zadok’s stage at Z75 and Z85 respectively for 14- and 30-dpa and snap frozen under liquid nitrogen. Developing caryopses from the spikes were selected, pulverized and stored at −80 °C in a deep freezer until further processing for RNA extraction. Total RNA from 35 cultivars at both 14 and 30dpa were isolated in duplicate as per the protocol reported previously [46]. Quantity and quality of isolated total RNA were determined using 2100 Bioanalyzer (Agilent Technologies, USA).

### Library preparation and NGS sequencing

Libraries for 31 samples from 14dpa and 32 samples from 30dpa stage (with 28 cultivars being common) were prepared and sequenced (100 bp paired-end reads) on an Illumina platform. Due to the lack of sufficient starting material data is not available for four cultivars, NW-93A, NW-108A, Pelada, and Vega at 14dpa, and three cultivars, Greece-25, NW-25A, and NW-51A at the 30dpa stage.

### Read assembly

Paired end-reads from raw sequence data were imported into CLC genomics workbench ver7.0.4 (CLC Bio, Denmark; presently Qiagen, USA) and all further computational analyses were performed with this tool unless otherwise stated. Imported reads were trimmed with default parameters. A quality check was performed both before and after trimming with default parameters. Trimmed reads were mapped against the DFCI *Triticum aestivum* Gene Index (TaGI) reference cDNA database consisting of 221,925 sequences (release 12.0, The Computational Biology and Functional Genomics Laboratory, Dana Farber Cancer Institute and Harvard School of Public Health).

### Transcriptome database during early and late grain-filling

The transcripts that were mapped against the TaGI reference database at early (14dpa) and late (30dpa) grain filling stages during wheat grain ontogeny were selected. From the mapped set of transcripts from the TaGI database, three sub-sets of transcript ids were created, common set for grain filling, unique set at 14dpa, and unique set at 30dpa. Functional annotation, enrichment analysis and R-fam analysis were performed through BLAST2GOv3.2 [31, 32] for the three subsets independently to identify the set of metabolic pathways that are unique to early and late grain filling stages and common ones that are involved in regulating various metabolic pathways across grain filling in wheat.

### Differential gene expression analysis and statistics

Sets of genes that are differentially expressed between 14dpa and 30dpa were identified through RNA-Seq experimentation. A false discovery rate (FDR) cut-off value of 0.01 was used to select the list of differentially expressed genes that were significant statistically from both mean based (Gaussian) and count-based (EDGE – Empirical analysis of Differential Gene Expression) statistics. Read expression values were normalized as RPKM (reads per kilobase per million) values and quantified in fold changes between two groups viz., 14 and 30dpa. Based on fold changes, the set of genes were grouped into four viz., U14 (uniquely expressed at 14dpa); D14 (differentially expressed in higher folds at 14dpa); D30 (differentially expressed in higher folds at 30dpa); and U30 (uniquely expressed at 30dpa) for further analyses (Fig. 1).

### Functional annotation and enrichment analysis for differentially expressed genes

A set of sequences from the four groups (U14 – 319; D14 – 16,237; D30 – 9740; U30 – 181) were retrieved from the TaGI database and blastx analyses were performed in parallel for the four groups against the non-redundant protein database in CLC genomics workbench ver7.0.4. Output files were converted into Blast2GO project files through a plug-in version and exported from CLC in “.dat” format. These files were then imported into Blast2GO Pro ver3.0.10 [31, 32] for functional annotation, enrichment and KEGG pathway mapping.

The four sequence sets were independently mapped and annotated with GO terms. Annotations were enriched using InterProScan option and followed by Run-Annex. GO-Slim analysis was performed to retain the annotations that are relevant to plant systems. Results of functional annotation were visualized using graphs and charts for the three major GO nodes viz., molecular function, biological process and cellular component independently. Simultaneously, the four annotated sequence sets were subjected to GO-enzyme code mapping to retrieve the pathway maps from KEGG supported by Blast2GO Pro ver 3.0.10. The list of metabolic pathways involving the differential expression of genes were grouped into nine categories based on metabolic networks between pathways and their types for interpretation of results.

## Additional files

Additional file 1: Genotype wise summary statistics for RNA sequencing reads at 14 and 30dpa. (XLSX 15 kb)

Additional file 2: Details of 319 transcripts with fold changes that are uniquely differentially expressed at 14dpa developmental stage across all the genotypes between 14 and 30dpa with FDR cut-off value at 0.01. (XLSX 380 kb)

Additional file 3: Details of 16,237 transcripts with fold changes that are differentially upregulated at 14dpa developmental stage across all the genotypes between 14 and 30dpa with FDR cut-off value at 0.01. (XLSX 28237 kb)

Additional file 4: Details of 9740 transcripts with fold changes that are differentially upregulated at 30dpa developmental stage across all the genotypes between 14 and 30DPA with FDR cut-off value at 0.01. (XLSX 17001 kb)

Additional file 5: Details of 181 transcripts with fold changes that are uniquely differentially expressed at 30dpa developmental stage across all the genotypes between 14 and 30dpa with FDR cut-off value at 0.01. (XLSX 213 kb)

Additional file 6: Nucleotide sequence details of 319 transcripts from TaGI cDNA database that are uniquely differentially expressed at the 14dpa developmental stage. (FA 249 kb)

Additional file 7: Nucleotide sequence details of 16,237 transcripts from TaGI cDNA database that are differentially upregulated at the 14dpa developmental stage. (FA 16754 kb)

Additional file 8: Nucleotide sequence details of 9740 transcripts from TaGI cDNA database that are differentially upregulated at the 30dpa developmental stage. (FA 9406 kb)

Additional file 9: Nucleotide sequence details of 181 transcripts from TaGI cDNA database that are uniquely differentially expressed at the 30dpa developmental stage. (FA 132 kb)

Additional file 10: Gene Ontology categorization and description for annotated transcripts (191) from 319 transcripts that are uniquely differentially expressed at the 14dpa. (XLSX 51 kb)

Additional file 11: Gene Ontology categorization and description for annotated transcripts (12792) from 16,237 transcripts that are differentially upregulated at 14dpa. (XLSX 3692 kb)

Additional file 12: Gene Ontology categorization and description for annotated transcripts (6513) from 9740 transcripts that are differentially upregulated at 30dpa. (XLSX 1553 kb)

Additional file 13: Gene Ontology categorization and description for annotated transcripts (75) from 181 transcripts that are uniquely differentially expressed at 30dpa. (XLSX 25 kb)

Additional file 14: Compilation of transcript IDs under various metabolic pathways across four groups viz., uniquely differentially expressed at 14dpa, differentially upregulated at 14dpa, differentially upregulated at 30dpa, and uniquely differentially expressed at 30dpa that are functionally annotated and KEGG pathway mapped metabolic pathways. (XLSX 253 kb)

Additional file 15: Metabolic pathways with highlighted enzyme ids that are linked with differentially expressed transcript IDs unique to 14dpa. (PPTX 641 kb)

Additional file 16: Metabolic pathways with highlighted enzyme ids that are linked with differentially expressed transcript IDs differentially upregulated at 14dpa. (PPTX 2573 kb)

Additional file 17: Metabolic pathways with highlighted enzyme ids that are linked with differentially expressed transcript IDs differentially upregulated at 30dpa. (PPTX 2498 kb)

Additional file 18: Metabolic pathways with highlighted enzyme ids that are linked with differentially expressed transcript IDs unique to 30dpa. (PPTX 671 kb)

Additional file 19: Supplementary details on various metabolic pathways differentially expressed between 14dpa and 30dpa. (DOCX 56 kb)

## Abbreviations

D14: Statistically differentially expressed transcripts that are upregulated during 14dpa
D30: Statistically differentially expressed transcripts that are upregulated during 30dpa
dpa: Days post anthesis
FDR: False discovery rate
KEGG: Kyoto encyclopedia of genes and genomes
NGS: Next-generation sequencing
RPKM: Reads per kilobase per million
TaGI: *Triticum aestivum* gene indices
U14: Statistically differentially expressed transcripts that are unique to 14dpa
U30: Statistically differentially expressed transcripts that are unique to 30dpa
