# Supplementary figures and images for: The transcriptome of the developing grain: a resource for understanding seed development and the molecular control of the functional and nutritional properties of wheat
Source: BMC Genomics. 2017 Oct 11;18:766. doi: 10.1186/s12864-017-4154-z (PMC5637334; doi:10.1186/s12864-017-4154-z)

## Slide 1
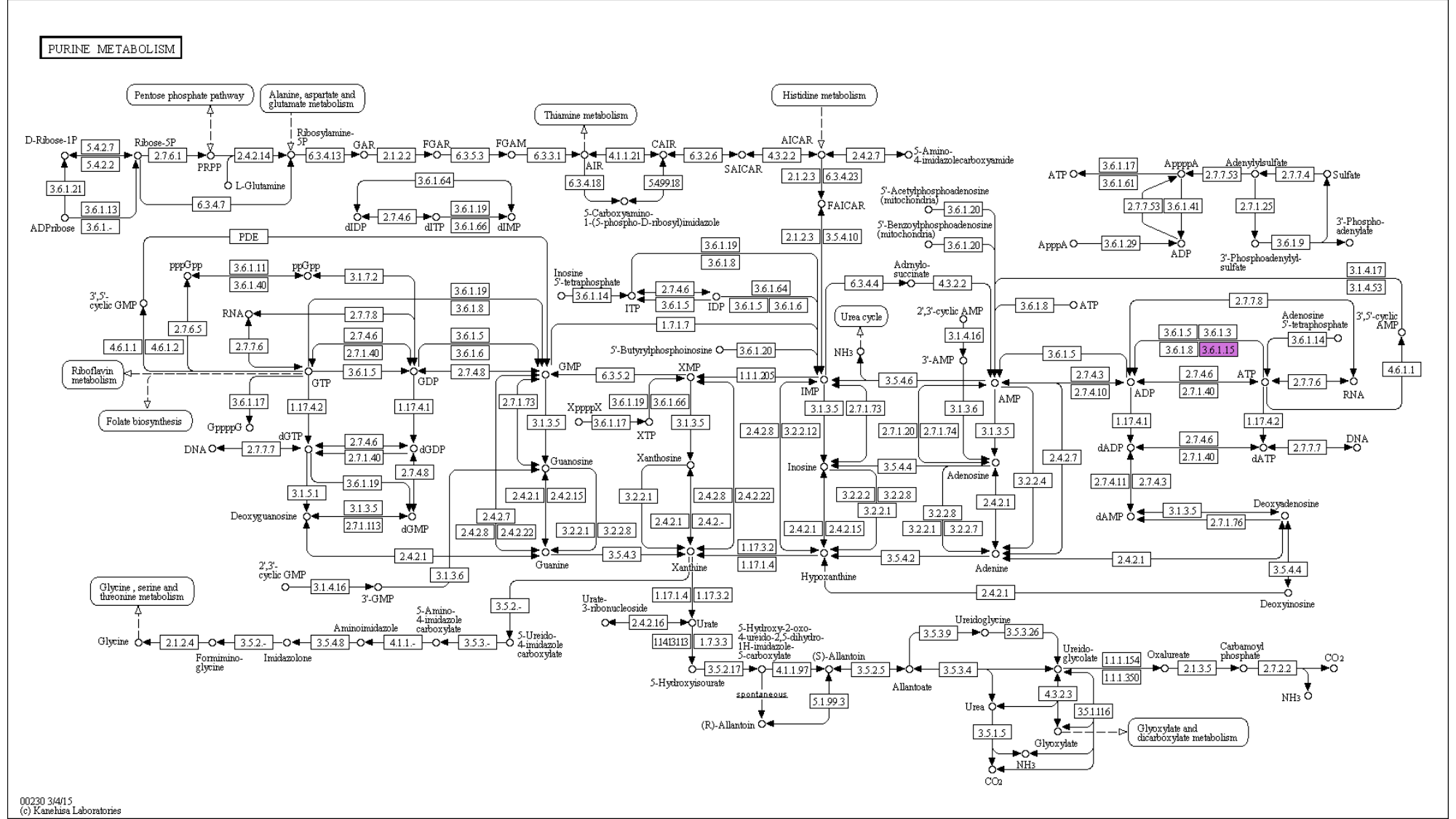

## Slide 2
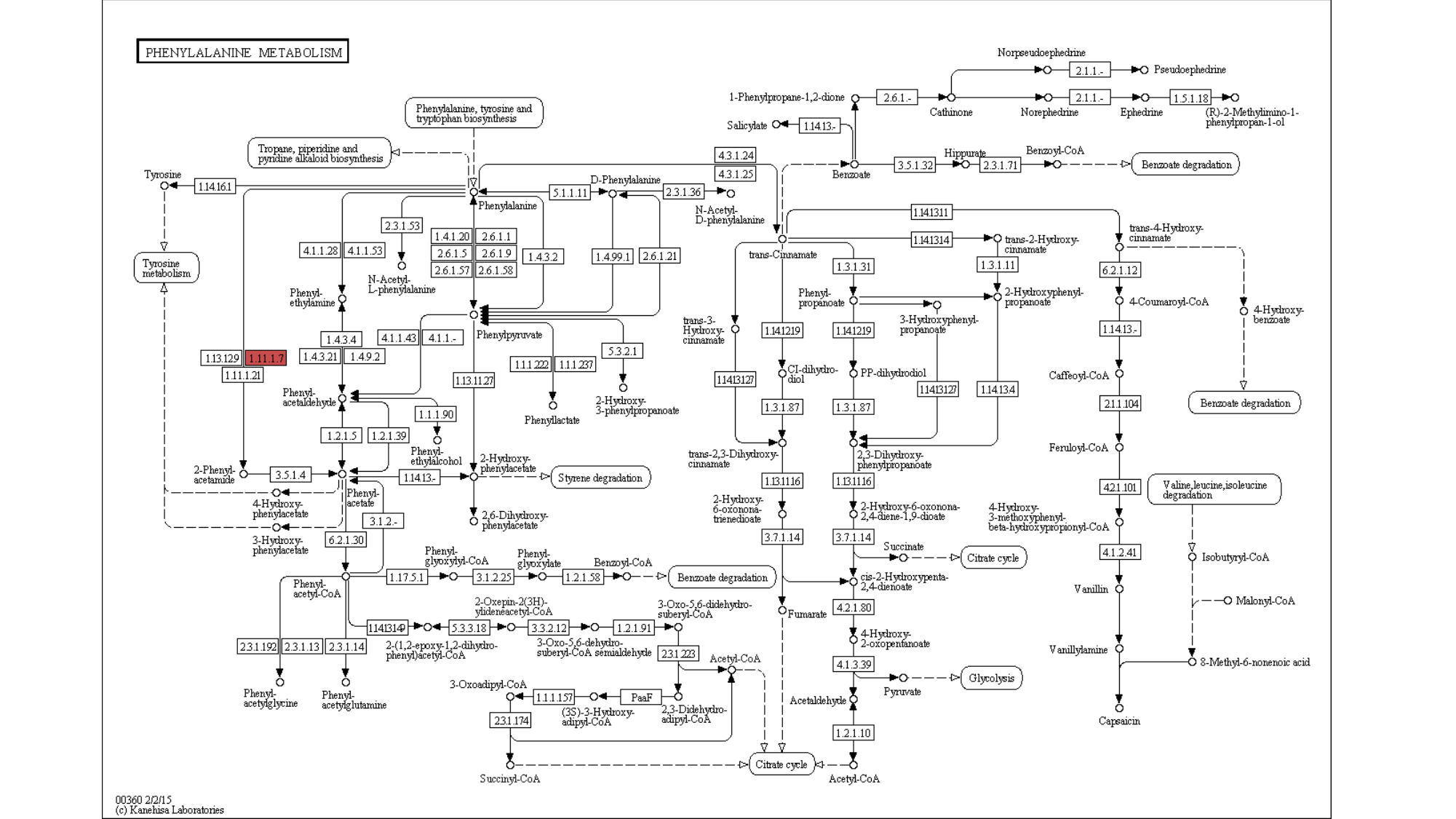

## Slide 3
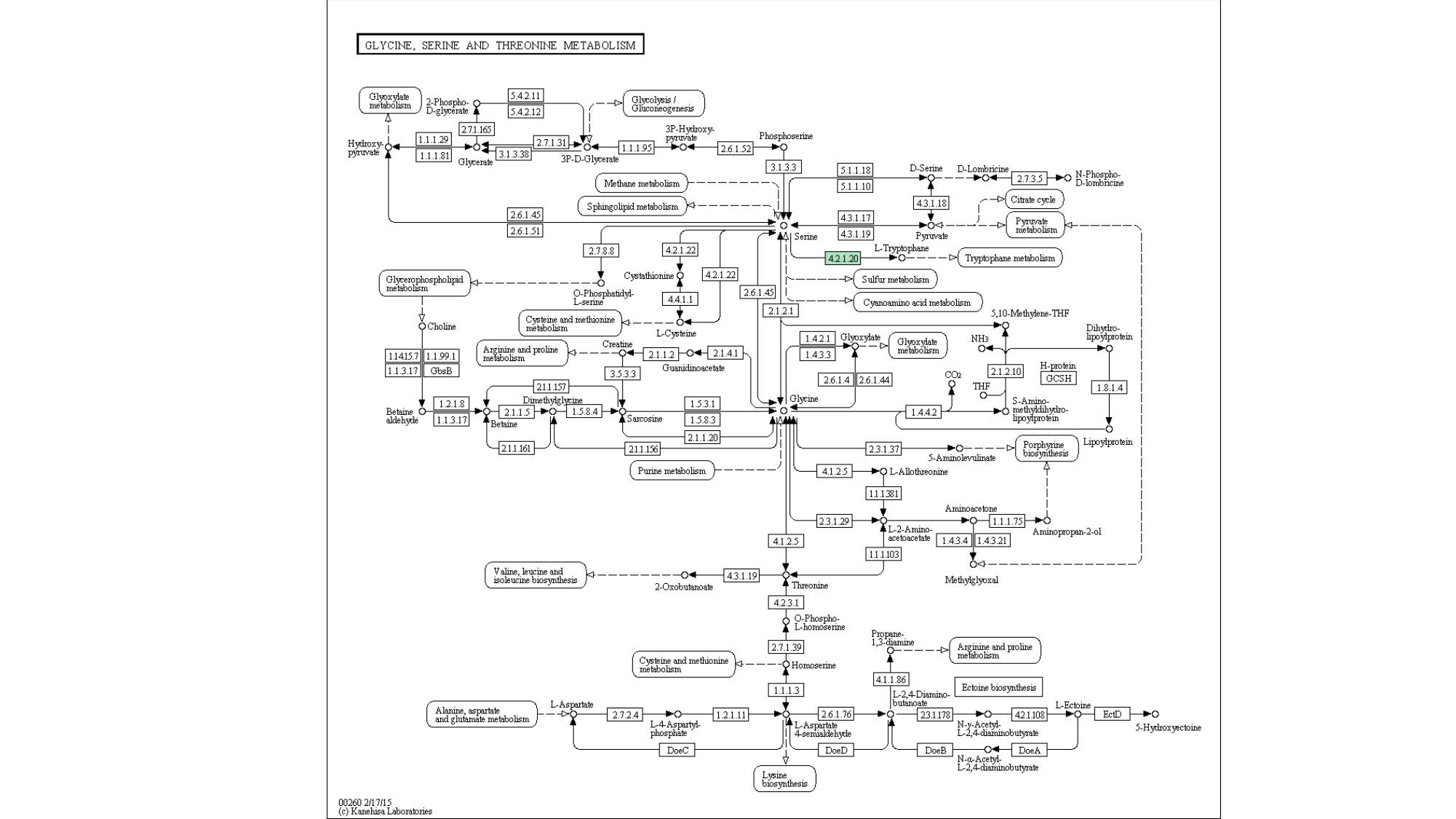

## Slide 4
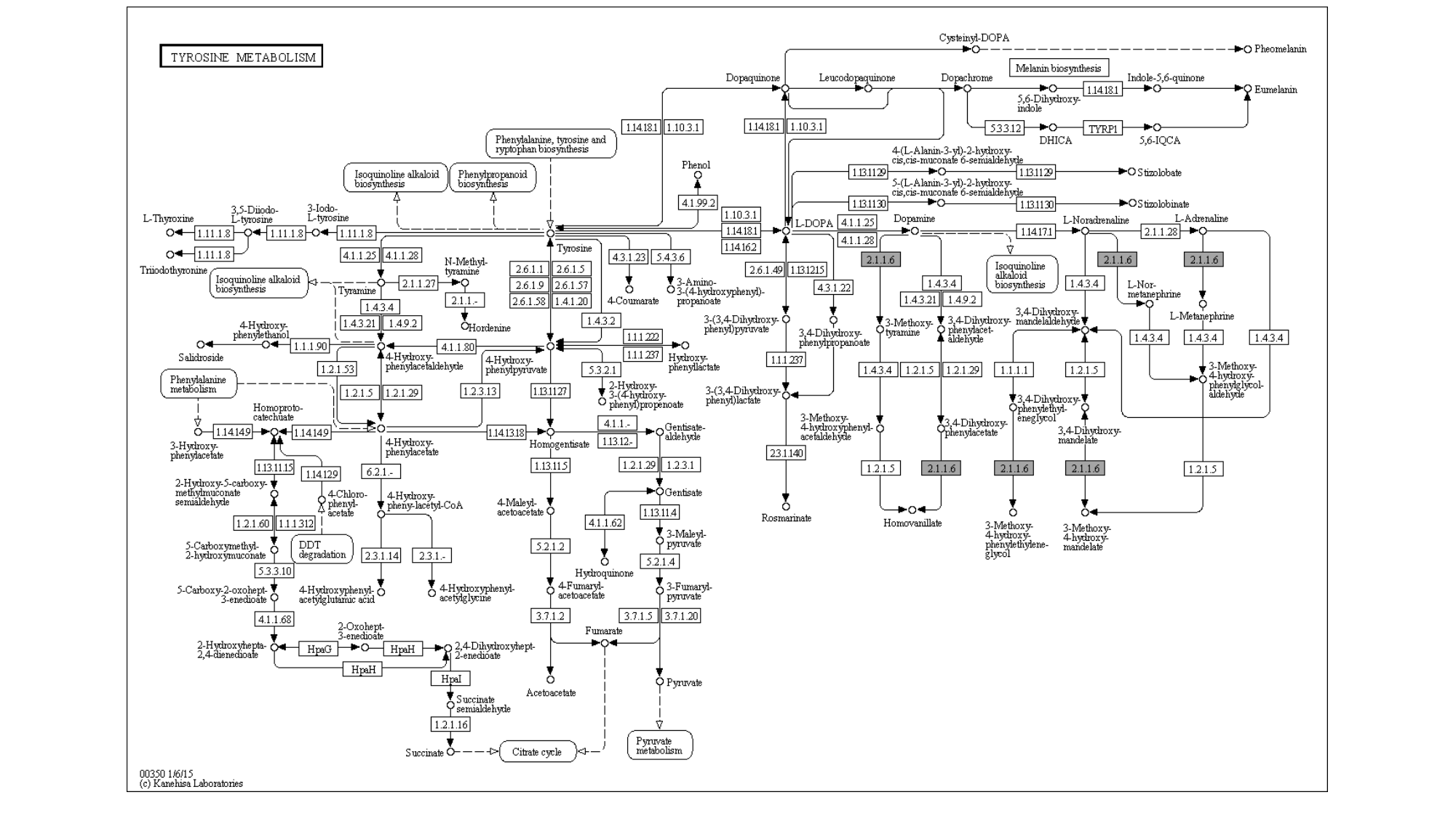

## Slide 5
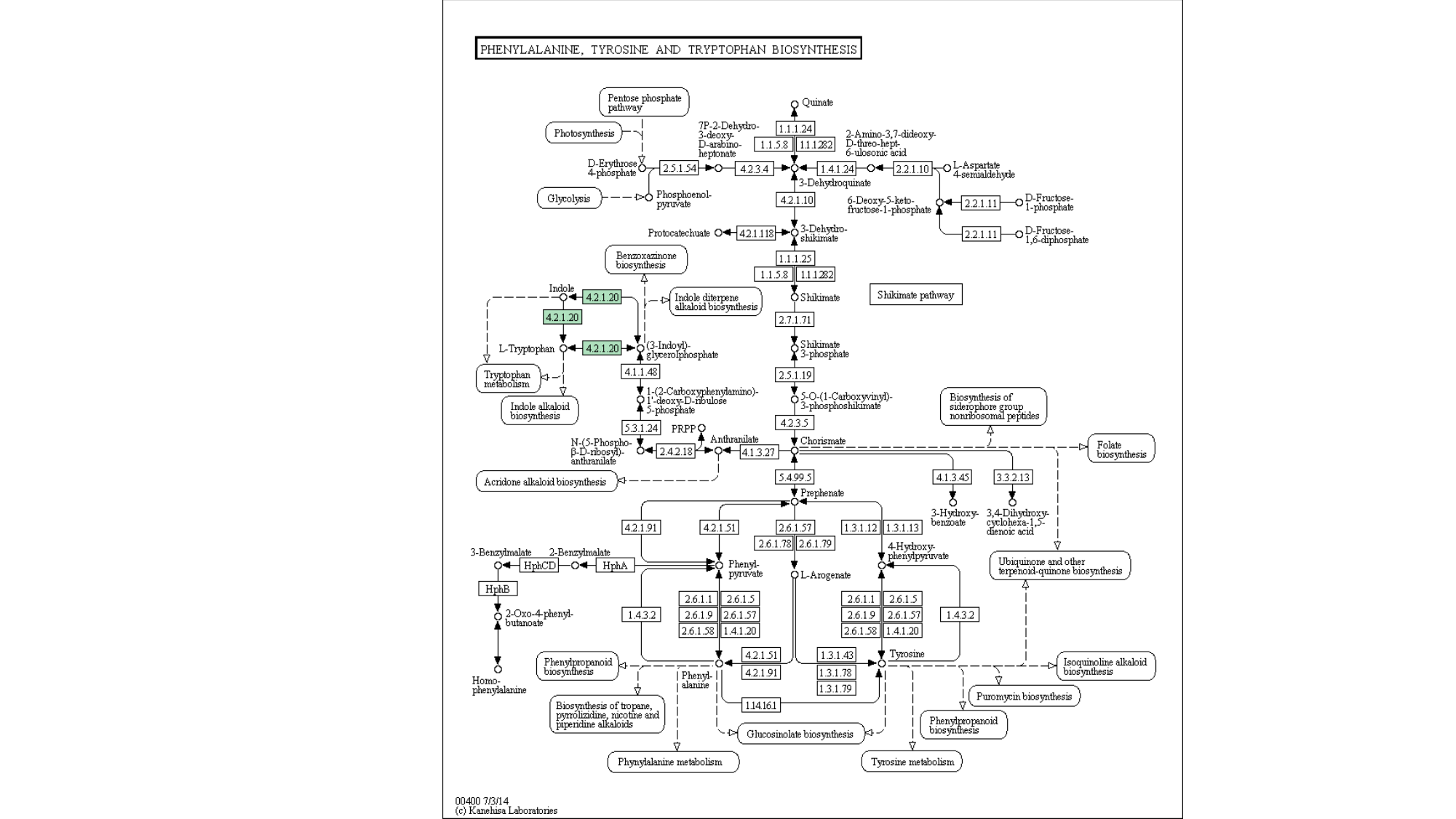

## Slide 6
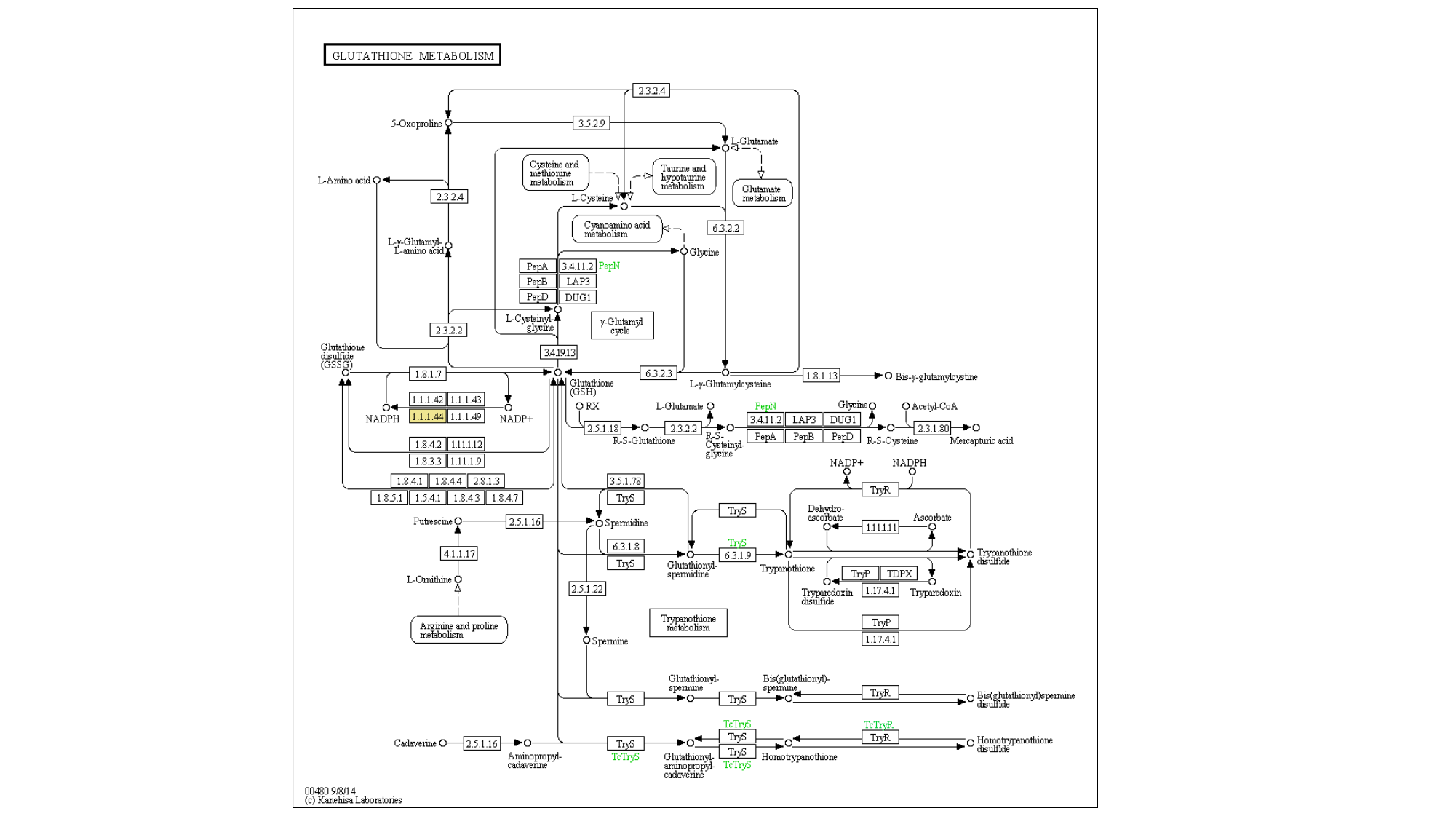

## Slide 7
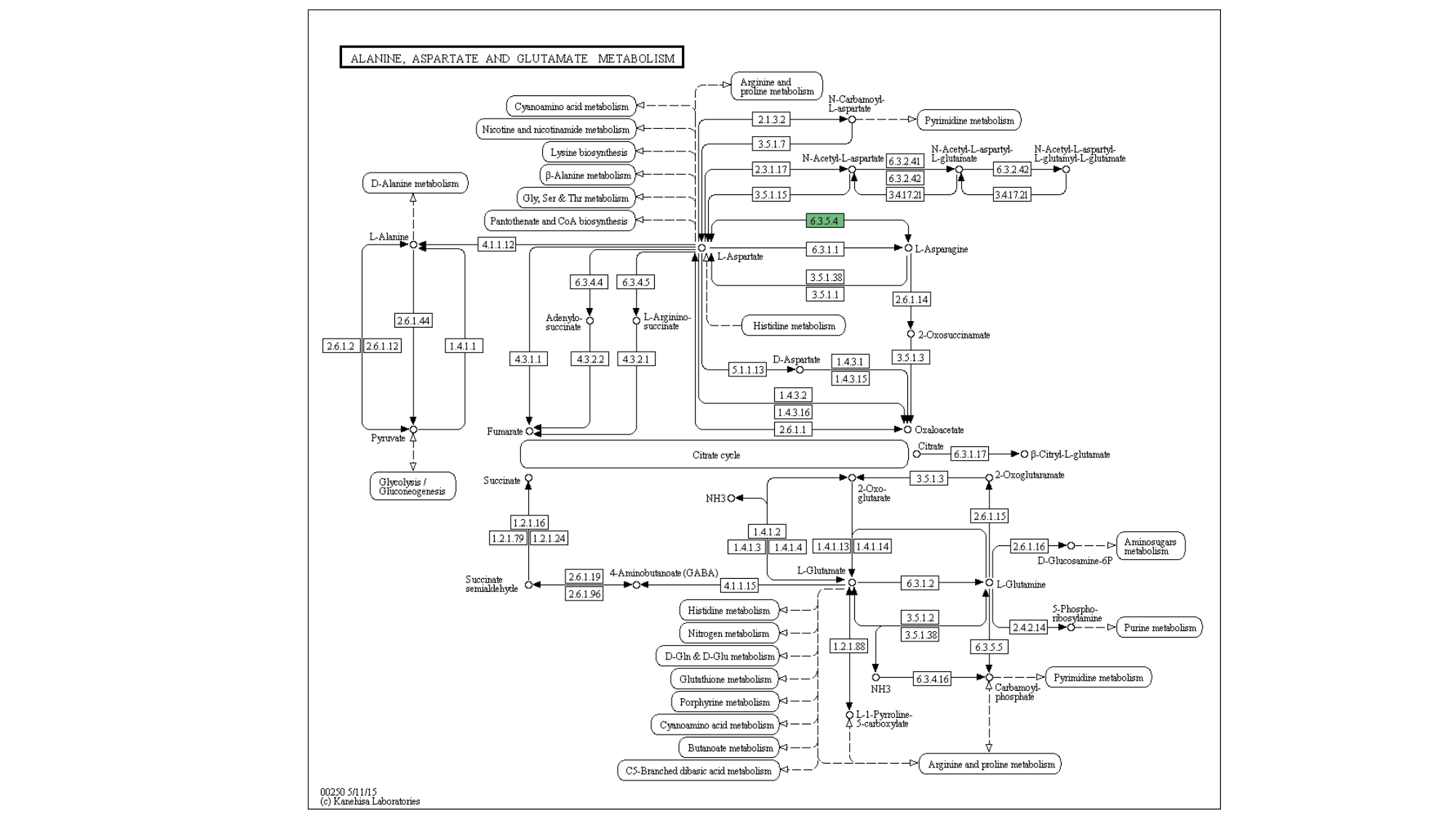

## Slide 8
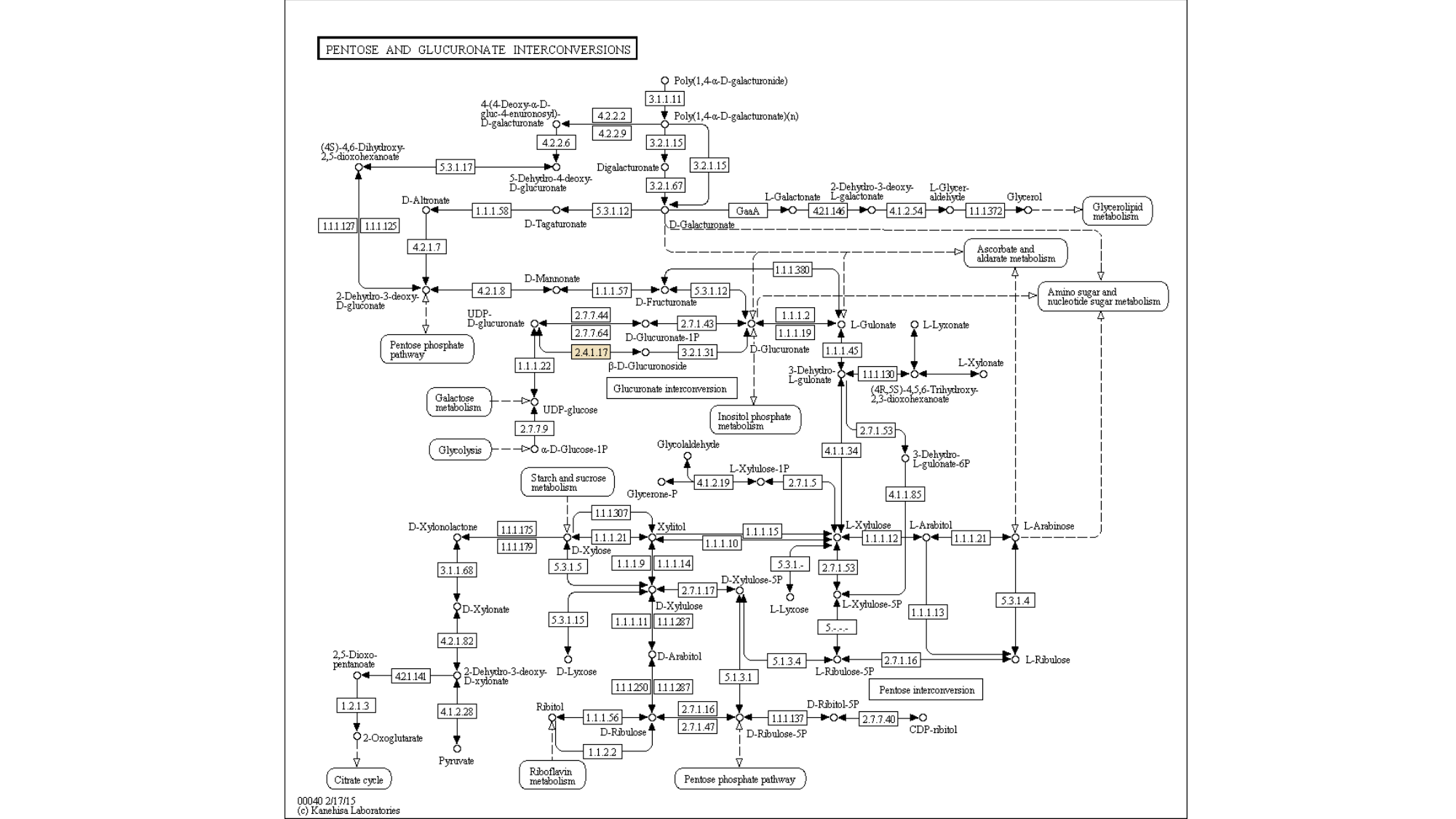

## Slide 9
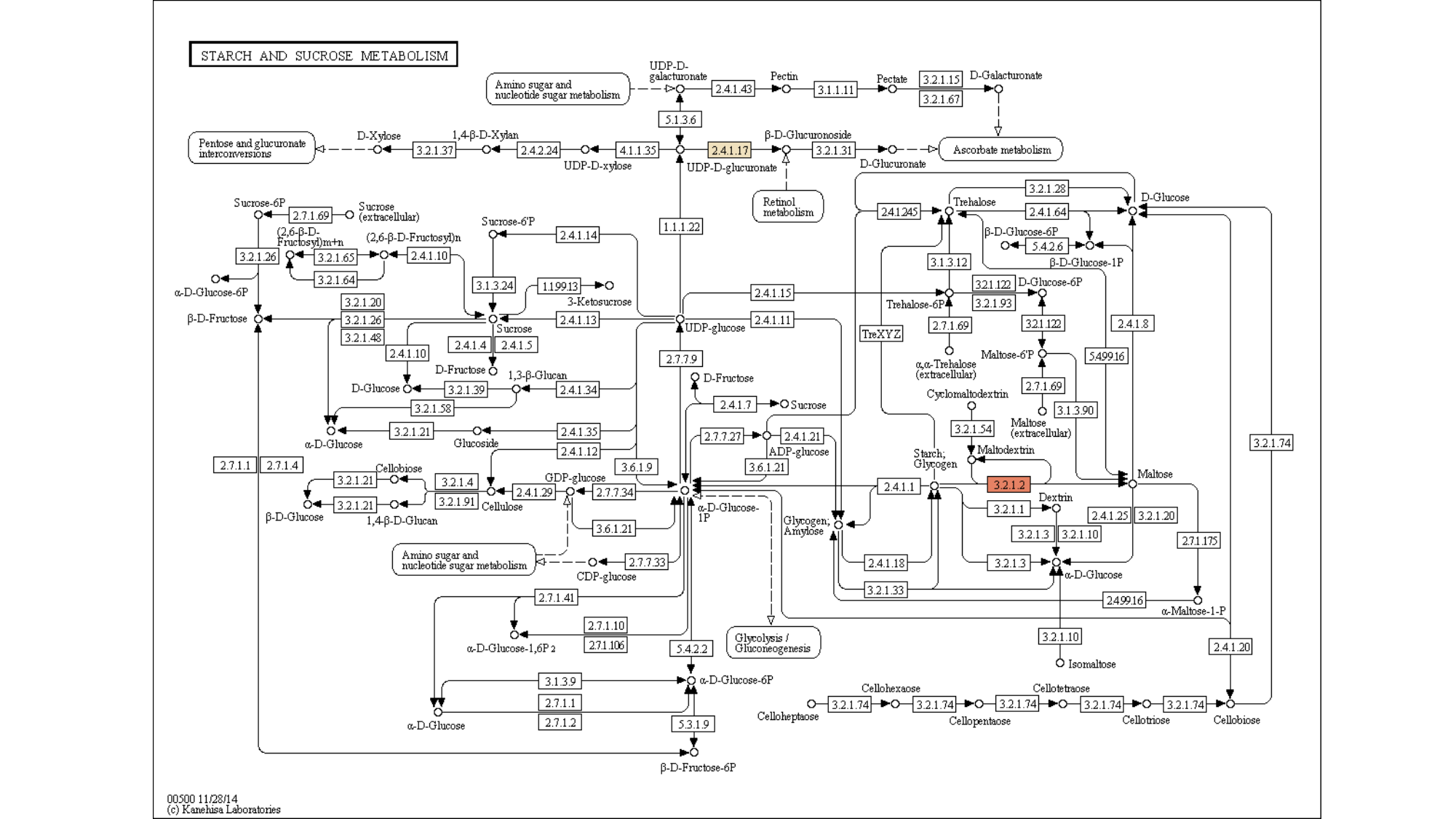

## Slide 10
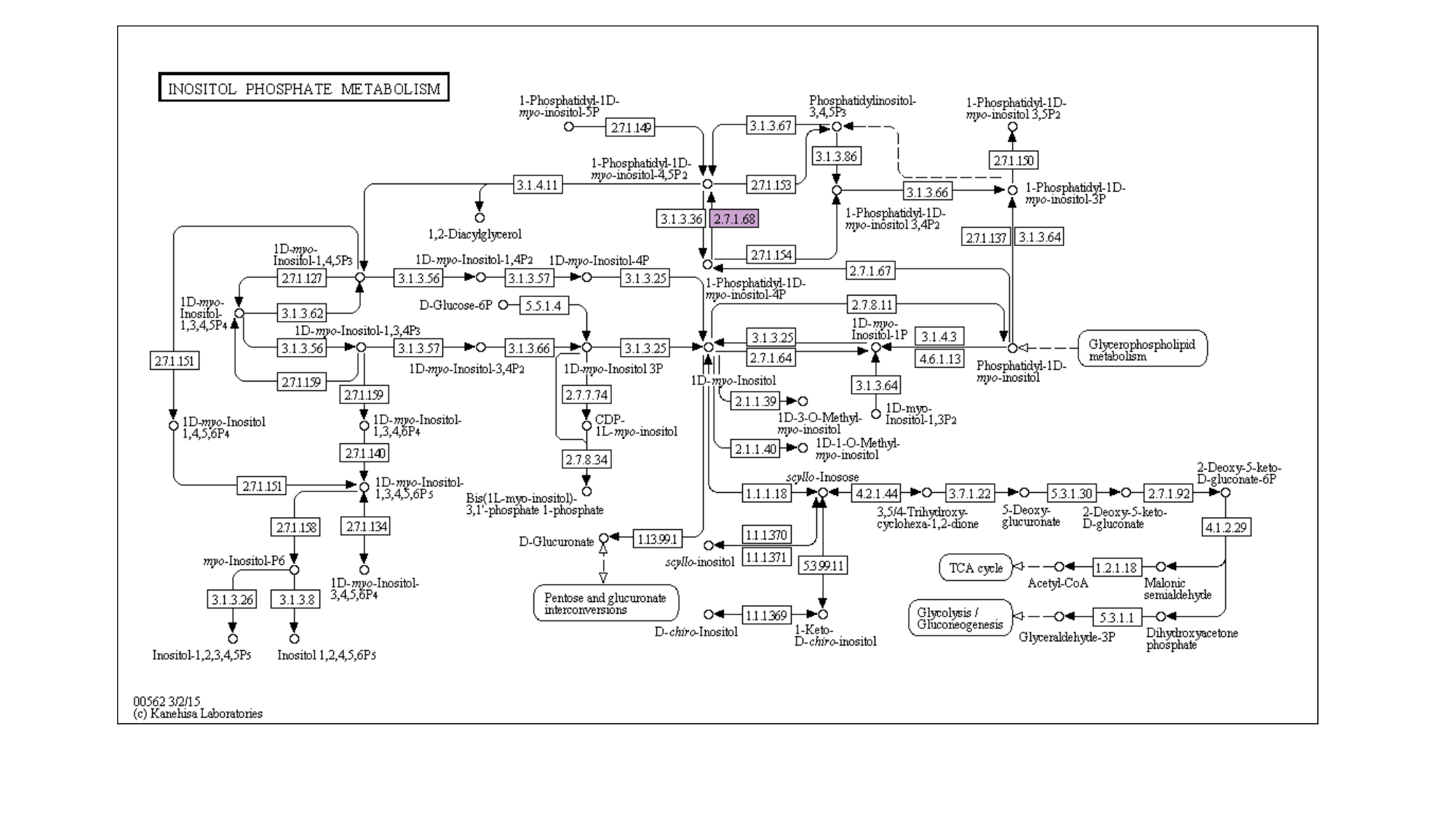

## Slide 11
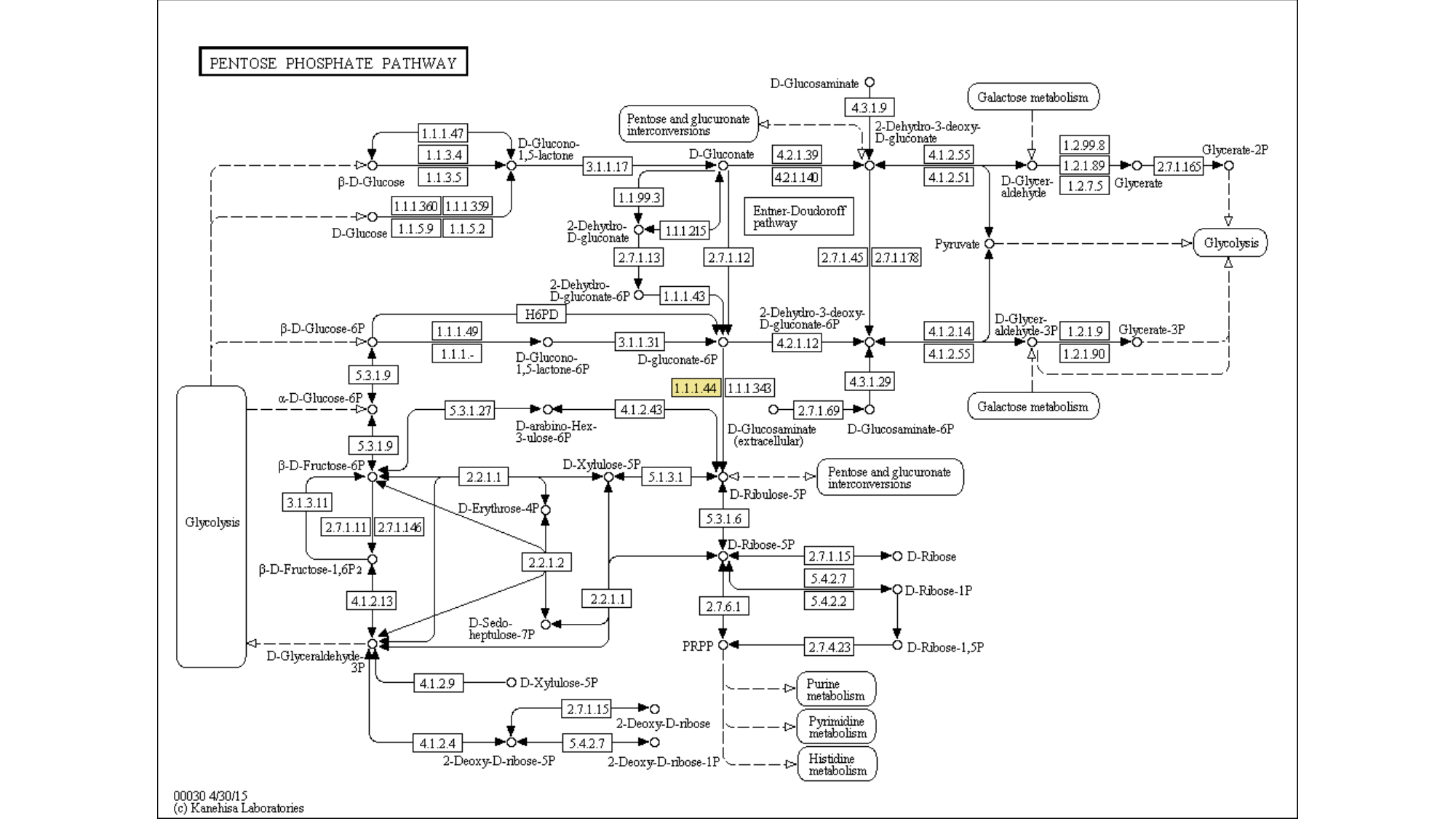

## Slide 12
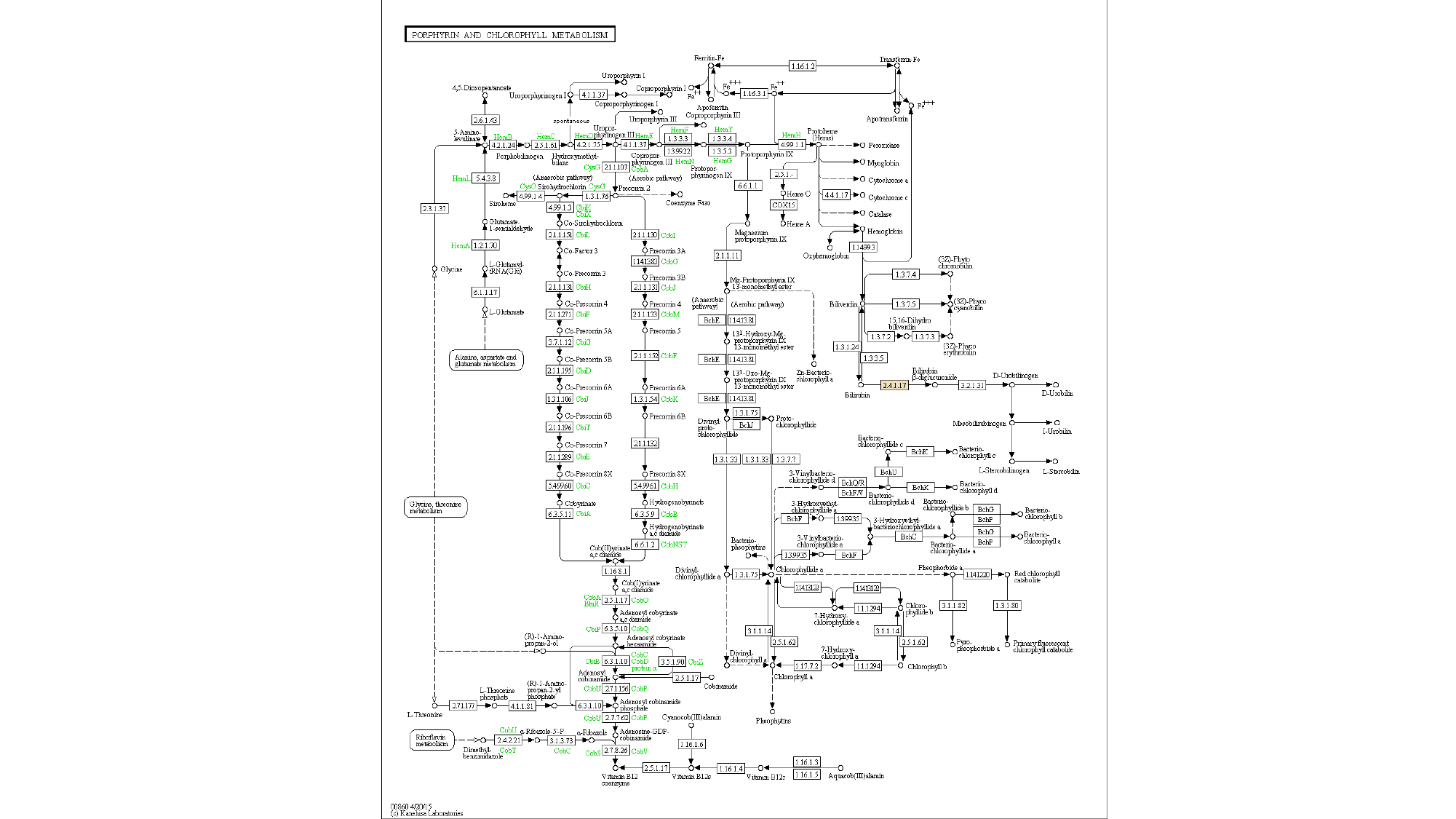

## Slide 13
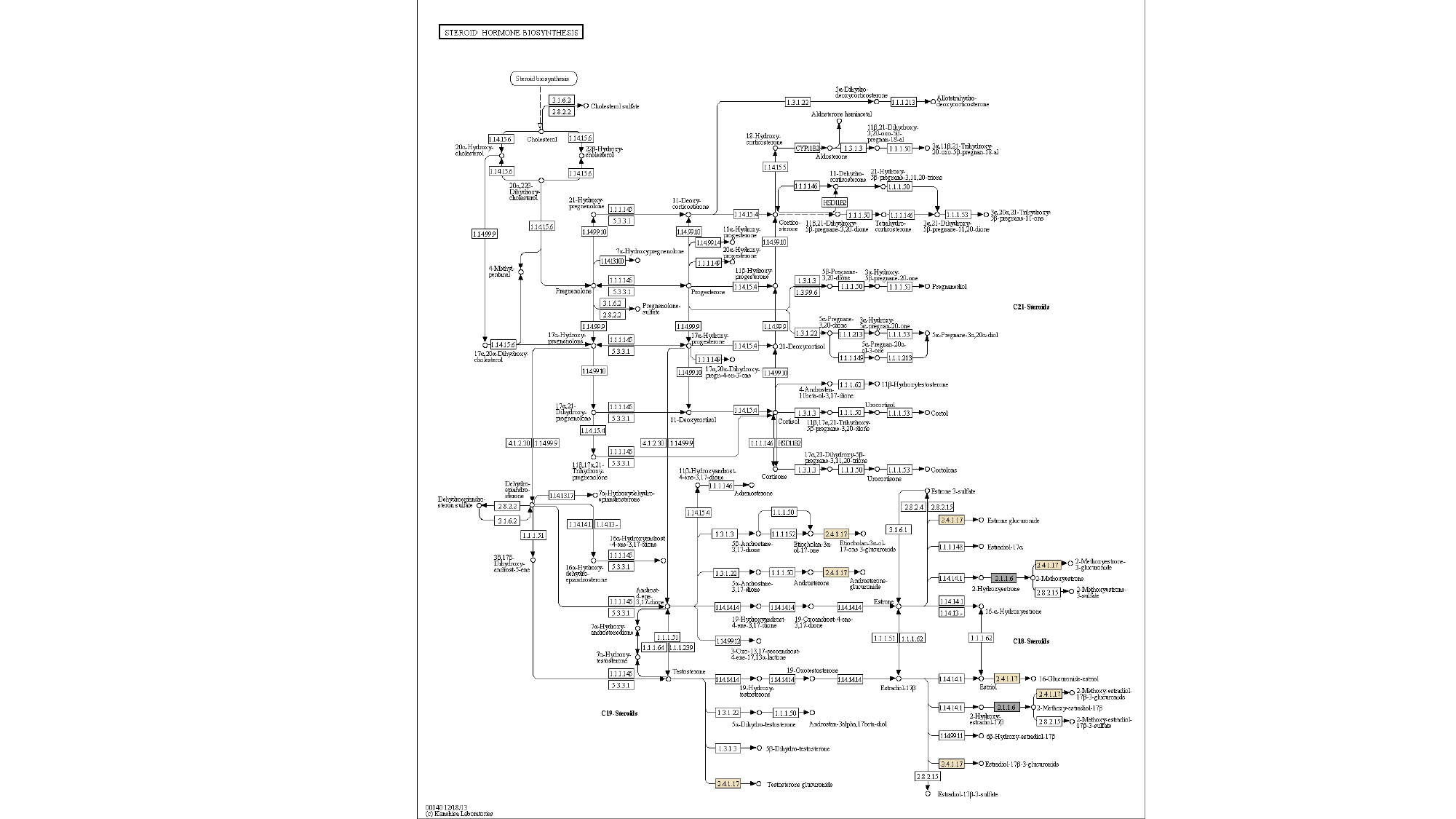

## Slide 14
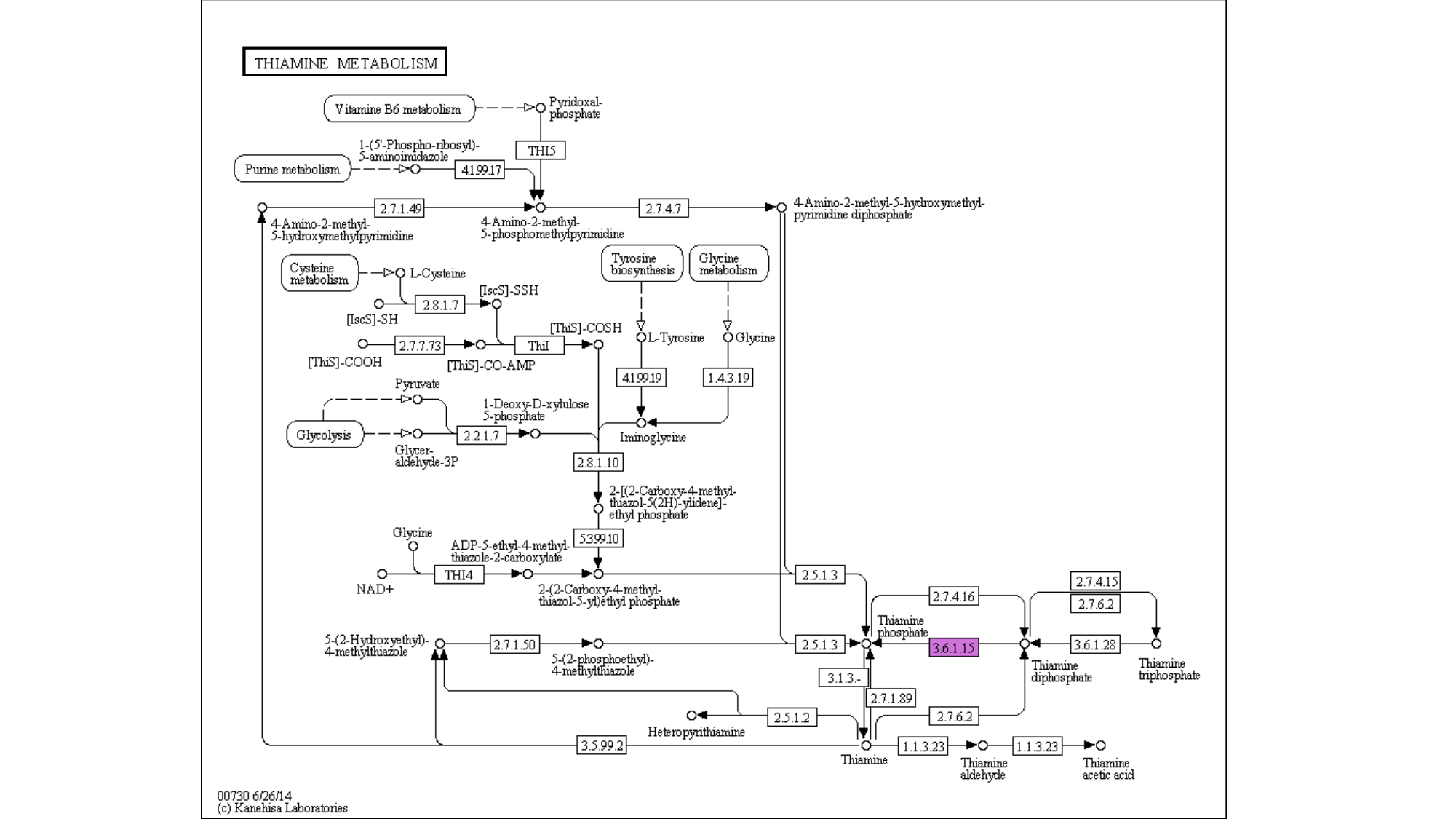

## Slide 15
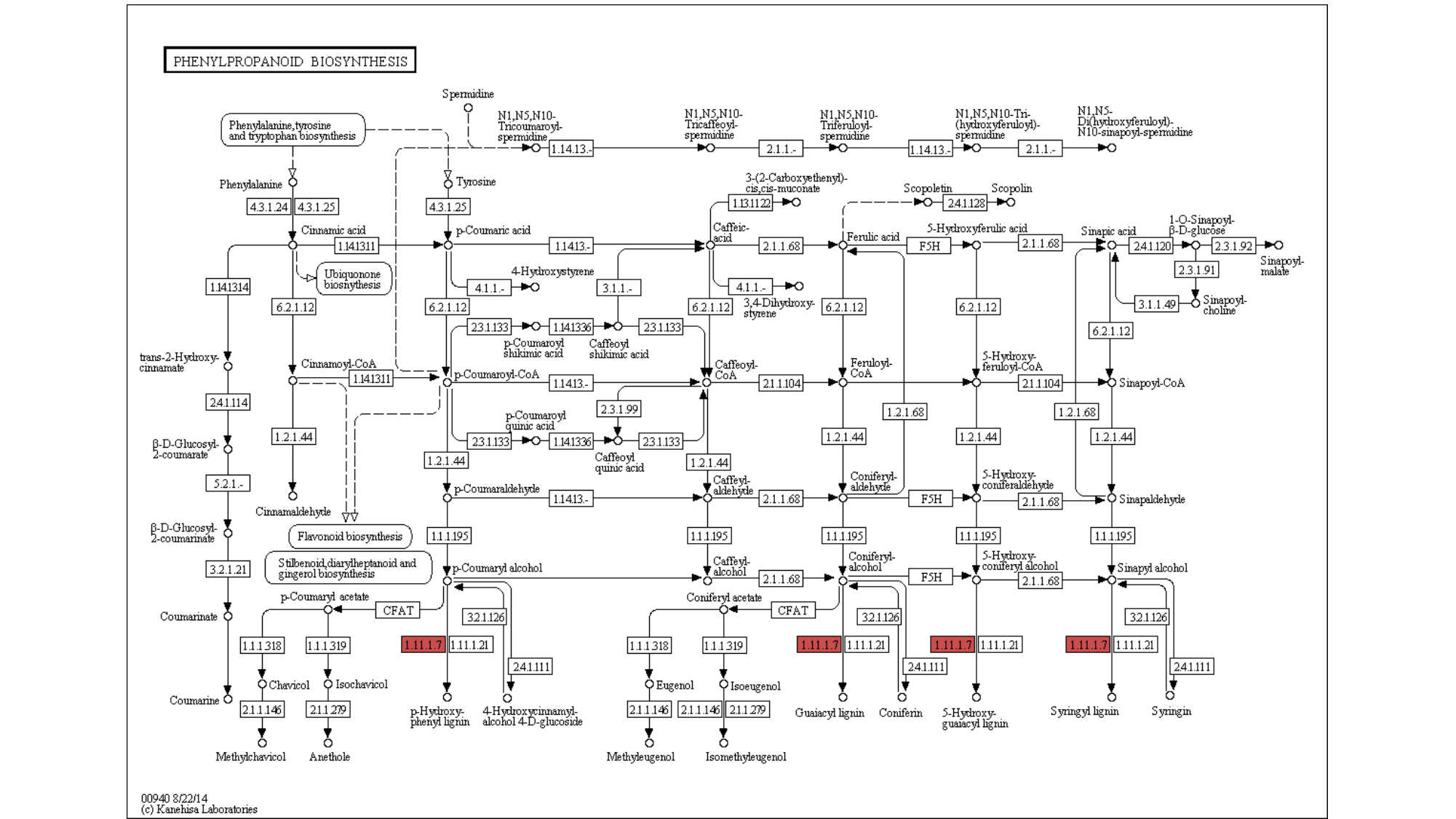

Supplement: Supplementary file 18 — Metabolic pathways with highlighted enzyme ids that are linked with differentially expressed transcript IDs unique to 30dpa. (PPTX 671 kb) [file 12864_2017_4154_MOESM18_ESM.pptx]
