# Supplementary material for: The transcriptome of the developing grain: a resource for understanding seed development and the molecular control of the functional and nutritional properties of wheat
Source: BMC Genomics. 2017 Oct 11;18:766. doi: 10.1186/s12864-017-4154-z (PMC5637334; doi:10.1186/s12864-017-4154-z)
Supplement: Supplementary file 19 — Supplementary details on various metabolic pathways differentially expressed between 14dpa and 30dpa. (DOCX 56 kb) [file 12864_2017_4154_MOESM19_ESM.docx]

**Supplementary details on various metabolic pathways differentially expressed between 14 and 30dpa.**

**Nucleotide metabolism:**

Three major pathways involved in nucleotide metabolism were: aminoacyl-tRNA biosynthesis, purine metabolism and pyrimidine metabolism (Additional file 14). Amino acylation of specific tRNA molecules with respective amino acids is accomplished through two classes of amino acid specific ligases (aminoacyl tRNA synthetase) [1, 2] based on recognition and identities between the cognate tRNA and the corresponding synthetase [3-8]. Besides direct acylation, aminoacyl-tRNAs are also synthesized through an indirect pathway – tRNA-dependent amino acid modification – especially for glutamine [1] as reflected in our analysis (Additional file 14; EC 6.3.5.7 and EC 6.1.1.17). The present study indicated that transcripts of synthetases specific for valine, isoleucine, tyrosine, histidine, phenylalanine, glutamine, cysteine, and proline were upregulated during the early part of grain filling (14dpa). Similarly, transcripts for aspartate, serine, lysine, threonine, glutamate and arginine specific synthetases were upregulated during the later part of grain filling and at maturity (30dpa). Alanine, leucine, glycine, and methionine specific synthetases are differentially expressed at 14 and 30dpa with different transcript IDs (Additional file 14). Further experiments are required to determine whether this is due to multiple copies [9] or sub-genome specific expression [10] or codon bias [1, 11] resulting in differential expression of different transcript IDs at 14 and 30dpa .

Monophosphates of inosine and triphosphates of uracil are the major precursors for purine and pyrimidine metabolism respectively [12]. Oxidized forms of triphosphates (dATP, dGTP, and dCTP) are formed through their respective diphosphates (dADP, dGDP and dCDP) and not directly from their nucleoside triphosphates (ATP, GTP, and CTP). While in the case of thymine, oxidized thymine nucleotide (dTMP) is formed from the oxidized form of uracil nucleotide (dUMP) which further acts as precursor to from dTDP and dTTP (Additional files 15-18). Polymerases (DNA and RNA) have transcript IDs that are different between their 14 and 30dpa stages indicating that there may be multiple copies or sub-genome specific or developmentally differential regulation patterns.

**Amino acid metabolism:**

There are 22 metabolic pathways involved in amino acid metabolism that are differentially regulated between 14 and 30dpa (Additional files 14-18). Amino acids play a major role both in protein constituent and in non-proteinaceous metabolism [13]. Nitrogen and sulfur metabolism are tightly linked and act as a backbone for most of the amino acid metabolic pathways. With respect to protein synthesis, the mechanistic target of rapamycin (mTOR) is an atypical serine/threonine protein kinase [14] that controls translational machinery and thereby regulats cell size through cell growth [15] through the mTOR signalling pathway. Here we focus mainly on differentially expressed genes between 14 and 30dpa that are involved in various metabolic pathways of protein associated amino acids.

The shikimate pathway connects photosynthesis and amino acid metabolism by utilizing photosynthates (erythrose-4-phosphate and phosphoenolpyruvate) to synthesize aromatic amino acids (phenylalanine, tyrosine and tryptophan) and has been reported to occur only in plants and microbes [16]. Differential expression of genes involved in the shikimate pathway specifically at 14dpa (Additional files 16, 17) coincides with the active accumulation of photosynthates. The shikimate pathway channels nearly one-fifth of the carbon fixed by plants and is especially involved in aromatic amino acid metabolism and the formation of secondary metabolites like quinones, folates, flavonoids, alkaloids, and lignin [16, 17] through various specialized metabolic pathways.

The aspartate family metabolic pathway plays a multifaceted role in plants [18] and involves glycine, serine and threonine metabolism; cysteine and methionine metabolism; and lysine metabolism [19]. With respect to monogastric animals, the aspartate family of amino acids constitute nearly 50% of the essential amino acids. Their biochemical importance in plants have been realized due to their metabolic expensiveness and thereby, they are mostly involved in inter-conversion process rather than various catabolic processes through which they are recycled and reused [19]. Transcriptional regulation of genes involved in aspartate-family amino acids’ metabolic pathways is not well known [20]. Most of the genes involved in glycine, serine and threonine metabolism are expressed at 14dpa (Additional files 14-18) indicates their activeness physiologically and biochemically. Glycine and serine pathways have been studied in much detail due to their oncogenic properties at hyper-activated conditions and their degradative pathway leads to one-carbon metabolism [21]. Although glycine degradation links with one-carbon metabolism, its involvement in photorespiration becomes expensive. Similar trends of differential expression at 14dpa were found in cysteine and methionine metabolism as in the earlier pathways (Additional files 16, 17). Cysteine and pyruvate is inter-converted at 14dpa, while at 30dpa, cysteine is converted to pyruvate for energy requirements (Additional files 16, 17). Methionine catabolism involves many intermediary steps leading to methionine salvage. Glutamate and cysteine results in glutamylcysteine which in turn combines with glycine to synthesize glutathione and is known to play a key role in plant chloroplasts with redox reactions, ascorbic acid metabolism [22], and during stress [23].

As with previously described metabolic pathways of the aspartate family; the branched chain amino acids valine, leucine and isoleucine biosynthesis were also differentially expressed at 14dpa (Additional files 16, 17). With respect to degradation pathways differentially expressed at 14dpa, leucine and valine was involved in replenishing propanoyl- and Acetyl-CoA respectively while isoleucine replenishes both. The leucine degradation pathway was additionally linked with terpenoid backbone biosynthesis. Aspartate and homoserine are the prime precursor for lysine biosynthesis and degradation pathways result in Acetyl-CoA and thereby are involved in the citrate cycle resulting in a balance between biosynthesis and energy production. Glutamate metabolism plays a central role in plant amino acid biosynthetic pathways with multiple functions like energy-releasing substrate, chlorophyll biosynthesis, synthesis of arginine and proline, γ-aminobutyric acid (GABA), and as structural determinant in proteins [24]. Degradation pathway of glutamate leads to various functional important metabolites like GABA involved in stress signalling, and the GABA-shunt resulting in light regulated higher metabolic flux through bypassing two steps of the citrate cycle[19]. Succinate semialdehyde – another intermediate involved in leaf patterning [25] and differentially expressed at 14dpa, while at 30dpa, succinate semialdehyde gets converted to succinate and linked with citrate cycle (Additional files 16, 17). Aspartate is the precursor for alanine biosynthesis with pyruvate being the degradation product of alanine that links with glycolysis. Besides aspartate, degradation of polyamine biosynthesis also results in the synthesis of alanine (Additional file 16, β-alanine metabolism). Alanine is also linked with cyanoamino acid metabolism through cyanoalanine and linked through aspartate and glutamate metabolic pathways [26].

Similar to other amino acid metabolism, genes involved in arginine and proline metabolism also differentially expressed at 14dpa (Additional files 16, 17). Glutamate is the prime precursor for both arginine and proline biosynthesis [27]. Proline biosynthesis involves either the acetylation route (Additional files 16, 17) through an intermediate ornithine and its transamination [28] or through non-acetylation without involvement of ornithine although involvement of ornithine has been reported [29, 30]. Proline plays a multifunctional role and is involved in regulating multiple biochemical pathways and developmental stages [27]. Arginine is synthesized from glutamate through ornithine via urea cycle [28]. Ornithine and arginine acts as a major precursor for polyamine biosynthesis and are known to be regulated during normal development and under stress [31].

In addition to regular amino acid metabolism, selenocompound metabolism too occur in wheat grains with differentially regulated genes at 14dpa (Additional files 16, 17). Selenium acts as an analog of sulfur and hence sulfur transporters are used by selenium and they form selenomethionine (SeMet) and selenocysteine (SeCys) in organisms through corresponding seleno-tRNAs [32]. Opal stop codon encodes for SeCys under the right mRNA context [32]. Although selenium is essential in trace quantities with many functional roles [33], it becomes toxic at even marginally higher concentrations [34] hence the window between necessity and toxicity is very narrow.

**Carbohydrate metabolism:**

Major carbohydrate metabolic pathways in which genes are differentially expressed between 14 and 30dpa are: fructose and mannose metabolism; galactose metabolism; pentose and glucuronate interconversions; starch and sucrose metabolism; inositol phosphate metabolism; glyoxylate and dicarboxylate metabolism; and amino sugar and nucleotide sugar metabolism (Additional files 14-18). Fructose, galactose, and mannose are phosphorylated through fructokinase, galactokinase (part of Leloir pathway) and hexokinase respectively [35-37]. Interconversion between fructosyl and glucosyl groups is differentially expressed at 30dpa indicating that at early stages photosynthesis results in maintaining sufficient glucose levels (Additional files 16, 17). Pentose and glucuronate interconversions can occur in any of the three ways*,* epimerization (when sugars have both UDP and uronic acid moieties), oxidation (to add uronic acid moiety using NAD^+^), and pyrophosphorylation through uridylyltransferase (to add UDP moiety) [38]. A network of nearly equal distribution patterns of differential expression (mutually exclusive) at 14 and 30dpa in pathways involved in starch and sucrose metabolism (Additional files 15-18). These network of pathways from starch and sucrose metabolism are linked with various other areas of carbohydrate metabolism [39] like pentose and glucuronate interconversions, amino sugars and nucleotide sugars metabolism, fructose and mannose metabolism, and galactose metabolism (Additional files 15-18). Unlike starch and sucrose metabolism, amino sugar and nucleotide sugar metabolism genes are differentially expressed more at 14dpa than at 30dpa (Additional files 16, 17). This pathway is tightly linked with pentose and glucuronate interconversions due to the requirement of UDP and uronic acid moieties especially for nucleotide sugars [38, 40]. Inositol phosphate metabolism is crucial in linking the carbohydrate metabolism with lipid metabolism (Additional files 16, 17). Inosito exists in nine stereoisomers with most common type is *myo*-inositol [41]. Although various reports indicate the possibility of ascorbate biosynthesis from inositol through *myo*-inositol oxygenase (MIOX) [40, 42], the metabolic pathway has not yet been deciphered. Glyoxylate and dicarboxylate metabolism links with major metabolic pathways like respiratory pathway, amino acid metabolism, and photosynthesis.

**Respiratory pathways:**

Metabolic pathways of a respiratory nature in which genes are differentially expressed between 14 and 30dpa are: glycolysis; citrate cycle; pentose phosphate pathway; pyruvate metabolism; oxidative phosphorylation; pantothenate and CoA biosynthesis; nicotinate and nicotinamide metabolism (Additional files 14-18). Most of the enzymes involved in glycolysis were differentially expressed both at 14 and 30dpa suggesting different genes of the gene family are responsible for the same functionality [43] and are active during the developmental process of the wheat grain (Additional files 14, 16, 17). It is surprising that the glycolytic pathway is the least studied among glycolysis, citrate cycle and electron transport chain [43]. Then glycolytic pathway is tightly linked with pathways of photosynthesis, carbohydrate metabolism, and other respiratory pathways like pentose phosphate pathway and citrate cycle. Pyruvate metabolism is the major link (in fact, prime precursor for the citrate cycle) between glycolysis and the citrate cycle. Differentially expressed genes from 14dpa dominate the citrate cycle pathway which continues from the end point of glycolytic pathway (Additional files 16, 17). Citrate cycle connects with various pathways of amino acid metabolism, fatty acid metabolism, and carbohydrate metabolism. Pentose phosphate pathway exhibits the differential expression pattern similar to glycolysis pathway (Additional files 16, 17). Although pantothenate is vitamin B_5_, Pantothenate and CoA biosynthesis was categorized under respiratory pathways due to the involvement and requirement of CoA in energy-yielding reactions. The pentose phosphate pathway is the major source of triose, pentose, hexose and heptose metabolic intermediates for various biosynthetic processes and thereby occupying a central role among respiratory pathways [44]. Oxidative phosphorylation is the crucial and most conserved pathway among all respiratory pathways involved in energy-yielding reactions. Most of the genes involved are differentially regulated at both 14 and 30dpa (Additional files 14, 16, 17) indicating the presence of different genes exhibiting the same enzyme function [43]. Vectorial translocation of protons in inward fashion is coupled with scalar reactions involving electron transfer and ATP synthesis [45]. Although mammalian mitochondria associates five major complexes (Complex I-IV, ATPase) for electron transfer and oxidative phosphorylation, two more complexes are involved in plant system *viz*., second Complex I, and an alternative oxidase (cyanide-insensitive) complex before ATPase [46]. Neither energy-yielding nor energy-consuming process can be completely functional without the presence of NAD and NADP, as key brokers in redox or energy metabolism and signalling pathways [47].

Although photorespiration is an important metabolic process in plants, KEGG pathway is generic in nature and is not tailor made for species or taxonomic groups [39] hence the pathways involving these metabolites were investigated for differential expression. Results indicate that metabolites that are involved in photorespiration were included as a part of glyoxylate and dicarboxylate metabolism under carbohydrate metabolism. In general, most of the genes involved in photorespiration were differentially expressed at 14dpa and serine to glycerate conversion might be a key regulatory pathway of photorespiration (Additional files 14, 16, 17). One carbon metabolism by a folate dependant pathway found under the heading vitamin metabolism is known to play a regulatory role involving glycine decarboxylase and serine hydroxymethyltransferase of the photorespiratory pathway and is tetrahydrofolate (THF) dependent [48].

**Photosynthesis:**

Occurrence of photosynthesis in wheat grains was reported based on enzyme and metabolite studies much earlier, in the 1970s [49, 50]. Genes that are differentially expressed between 14 and 30dpa and involved in photosynthetic pathways are: photosynthesis; porphyrin and chlorophyll metabolism; and carbon fixation in photosynthetic organisms (Additional files 14, 16, 17). All these three pathways were expressed specifically at 14dpa indicating that photosynthesis was more active during the grain filling process in order to fix carbon for increased grain yield (Additional files 16, 17). Tetrapyrroles act as the major intermediates in porphyrin and chlorophyll metabolism with almost 27 genes [51] required to synthesize chlorophyll that harvests light energy [52]. The presence of all the six genes of the NAD-ME type C_4_ pathway in developing wheat grain at 14dpa with C_4_ specific copies indicated the possibility of C_4_ photosynthesis (Additional file 16). The presence of 4 carbon compounds specifically in wheat grains [53] (absent in leaves) helped to substantiate our discovery of the C_4_ pathway without Kranz anatomy specifically in the grains of wheat – a well-known C_3_ plant – and this outcome with a proposed model has been reported elsewhere [54]. We termed the anatomy of C_4_ photosynthesis being accomplished in a non-Kranz anatomical fashion as “Bose anatomy” in honour of his early findings on the use of 4C compound for photosynthesis in 1920’s in *Hydrilla* sp., which later found to be of single-cell C_4_ photosynthesis type [55].

**Lipid metabolism:**

Lipids are an important component of a cell and are involving in multiple biological processes with plants producing the majority of the world’s lipids [56]. Based on chemical structure and their derivatives, lipids are classed into eight groups *viz*., fatty acyls, glycerolipids, glycerophospholipids, sphingolipids, sterol lipids, prenol lipids, saccharolipids, and polyketides and a 12-digit unique identifier for lipid molecule were assigned [57]. Fatty acid biosynthesis; fatty acid elongation; fatty acid degradation; biosynthesis of unsaturated fatty acids; linoleic acid metabolism; α-linolenic acid metabolism; arachidonic acid metabolism; butanoate metabolism; glycerolipid metabolism; glycerophospholipid metabolism; sphingolipid metabolism; ether lipid metabolism; and phosphatidylinositol signalling system pathways involve genes that are differentially expressed between 14 and 30dpa (Additional files 14-17). Genes involved in biosynthesis-, elongation-, and degradation- of fatty acids, biosynthesis of unsaturated fatty acids, and sphingolipid metabolism are differentially expressed at 14dpa in higher folds (Additional files 14, 15). Linoleic acid-; α-linolenic acid-; arachidonic acid-; and ether lipid- metabolism involves different set of genes that are differentially expressed during 14 and 30dpa for the same enzymatic reaction (Additional files 14, 15). Butanoate-; glycerolipid-; glycerophospholipid-metabolism and phosphatidylinositol signalling system involves genes that are differentially expressed either at 14 or at 30dpa in accordance with the pathways they are linked with (Additional files 14, 15).

**Hormone biosynthesis:**

Different hormones play an essential role during different developmental stages or in different organs in plants to maintain the growth cycle. Traditionally, in plants five hormones were known *viz*., auxins, gibberellins, cytokinins, ethylene and abscisic acid and are commonly known as plant growth regulators [58] with the role of steroids in plants defined much later, especially brassinosteroids [59, 60]. Various hormone biosynthetic pathways involving genes that are differentially expressed between 14 and 30dpa were steroid-; steroid hormone-; and zeatin- biosynthesis (Additional files 14, 16, 17). Involvement of different genes at 14 and 30dpa in steroid and steroid hormone biosynthetic pathways indicates an altered gene (or sub-genome) expressed pattern for same enzymatic reactions. Although the pathways were generated from KEGG (Additional files 16, 17) depicting generic ones; functional annotation (Additional files 11, 12) indicates the resemblance of the enzymes in steroid hormonal biosynthesis from non-plant systems to plants and may be useful in charting out the detailed metabolic pathway in future for steroid hormones in plants specifically.

**Vitamin metabolism:**

Although vitamins are named so due to the lack of ability to synthesize in them in humans, they are also essential for plants due to which the metabolism exists in plants. Vitamins exhibit their redox potential nature and functionally acts as cofactors, and antioxidants in plants [61]. Among the vitamin metabolic pathways (B_1_, B_2_, B_6_, B_7_ and B_9_) in which the genes are differentially expressed between 14 and 30dpa, only vitamin B_1_ (Thiamine metabolism) and B_9_ (folate biosynthesis) metabolic pathways were differentially expressed during 30dpa. While genes for other pathways, B_2_ (riboflavin metabolism), B_6_ (pyridoxine metabolism), and B_7_ (biotin metabolism) are differentially expressed at 14dpa (Additional files 14-18). Involvement of riboflavin metabolism in a novel signalling pathway for systemic resistance in plants during biotic [62] and abiotic [63] stresses has been reported. One carbon pool metabolism by a folate dependant pathway involves one carbon transfer reactions mediated by tetrahydrofolate (THF) cofactors [64]. Tetrahydrofolate (THF) constitutes a pterin ring, a p-aminobenzoic acid, and a poly-Glu chain with variably up to eight residues collectively known as folate that mainly converts one carbon units from their reduced state to an oxidized state [64]. Most of the genes involved in one carbon pool are differentially expressed at 14dpa (Additional files 14, 16, 17). Reports also indicate their regulatory role through the THF dependent enzymes glycine decarboxylase and serine hydroxymethyltransferase that are involved in the photo respiratory pathway [48]; refer to the heading respiratory pathways for details. Taurine and hypotaurine is an acid containing amine group but not an amino acid [65] and the metabolic pathway has been categorized under vitamin metabolism (although some report this as a conditionally essential amino acid [66]) due to its deficiency symptoms in animal systems [67, 68]. Taurine is reported to play major functions like osmoregulation, detoxification, membrane stabilization, and modulating cellular calcium levels [66]; with minimal reports in plants of hypotaurine, an intermediate of taurine exhibiting antioxidant potential [69]. The present analysis reveals different genes (or sub-genome) are differentially expressed at 14 and 30dpa for the same enzymatic reactions involving taurine and hypotaurine metabolic pathways (Additional files 14, 16, 17). Research on taurine and hypotaurine metabolic pathways is in its infancy and its importance in animal system reflects the need for more focus and attention in plant systems in future.

**Specialized metabolism**

Based on their functional importance in plants produced during stress or in specialized tissues or even at specific developmental stages; and their biological value, “specialized metabolism” was considered more appropriate than “secondary metabolism” [70, 71]. Genes involving specialized metabolic pathways that are differentially expressed between 14 and 30dpa are: phenylpropanoid-; terpenoid backbone-; ubiquinone and other terpenoid-quinone-; carotenoid-; diterpenoid-; flavone and flavonol-; flavonoid-; and benzoxazinoid- biosynthesis (Additional files 14-18). The phenylpropanoid biosynthetic pathway with its core structure from shikimate pathway with phenylalanine and tyrosine being the major precursors, generates an array of diverse specialized metabolites through enzyme superfamilies like oxygenase, ligase, oxidoreductase and transferases [72]. Metabolites synthesized from phenylpropanoid biosynthesis regulated through MYB (V-Myb avian Myeloblastosis viral oncogene homolog) transcription factor [73] acts as precursors for various other specialized metabolic pathways like flavonoids and lignins (Additional files 15-18). Genes involved in flavonoid biosynthesis were differentially expressed both at 14 and 30dpa indicating the role of different genes (or sub-genome) are expressed differentially at different developmental stages for the same enzymatic reactions (Additional files 16, 17). Regulation of flavonoid biosynthesis at the transcriptional level [74] for pigmentation [75] and during stress [76] has been reported. The pigmentation in wheat grains indicates, part of flavonoid biosynthesis (especially anthocyanin biosynthesis) may be differentially expressed with tissue specificity (aleurone or pericarp) for blue or purple or red colour [77-79]. Most of the genes involved in terpenoid backbone biosynthesis and ubiquinone and other terpenoid-quinone biosynthetic pathways were differentially expressed at 14dpa (Additional files 16, 17). Terpenoid backbone biosynthesis occurs through alternate pathways with cytosolic mevalonate (MVA) pathway and plastidic 1-deoxy-D-xylulose-5-phosphate (DOXP) pathway [80] to produce isopentenyl diphosphate (IPP) [81]. Metabolites from terpenoid backbone biosynthesis act as a major precursor for zeatin, monoterpenoid, diterpenoid, carotenoid, sesquiterpenoid, diterpene alkaloids, and also for terpenoid-quinones like plastoquinone, tocopherol (Additional files 16, 17). Metabolites like plastoquinones and phylloquinones (Vit K_1_) – terpenoid-quinones – from ubiquinone and other terpenoid-quinone biosynthetic pathway act as the major electron and proton carriers in energy production machinery [82]. Ubiquinone and other terpenoid-quinone biosynthetic pathways involve cinnamate and coumarate from phenylpropanoid biosynthesis to synthesize ubiquinone (Additional files 16, 17). Carotenoids and diterpenoids are the major biosynthetic pathways that involve metabolites from terpenoid backbone biosynthesis with the genes involved being differentially expressed at 14dpa. Carotenoid biosynthetic pathway [83-85] generates lycopene, xanthins, and abscisic acid; while the diterpenoid biosynthetic pathway [86, 87] mainly generates gibberellic acids [88] (Additional files 16, 17). Benzoxazinoids are alkaloids especially 2,4-dihydroxy-1,4-benzoxazin-3-one (DIBOA) with protective and allelopathic effect synthesized in selective taxa of Poaceae (Pooideae and Panicoideae) and some solitary species in dicots (Ranunculaceae, Lamiaceae and Plantaginaceae) [89]. Genes involving the benzoxazinoid biosynthetic pathway are differentially expressed at 30dpa authenticating their protective mechanism and serve as a natural defense against insect pests, fungi and other pathogens (Additional files 16-18).

References:

1. Woese CR, Olsen GJ, Ibba M, Söll D: **Aminoacyl-tRNA synthetases, the genetic code, and the evolutionary process.** *Microbiology and Molecular Biology Reviews* 2000, **64:**202-236.

2. de Pouplana LsR, Schimmel P: **Aminoacyl-tRNA synthetases: potential markers of genetic code development.** *Trends in biochemical sciences* 2001, **26:**591-596.

3. Schulman LH: **Recognition of tRNAs by aminoacyl-tRNA synthetases.** *Prog Nucleic Acid Res Mol Biol* 1991, **41:**23-87.

4. Cavarelli J, Moras D: **Recognition of tRNAs by aminoacyl-tRNA synthetases.** *The FASEB journal* 1993, **7:**79-86.

5. Weil J, Parthier B: **Transfer RNA and aminoacyl-tRNA synthetases in plants.** In *Nucleic Acids and Proteins in Plants I.* Springer; 1982: 65-112

6. Lea P, Norris R: **tRNA and aminoacyl-tRNA synthetases from plants.** *Phytochemistry* 1972, **11:**2897-2920.

7. Jakubowski H, Fersht AR: **Alternative pathways for editing non-cognate amino acids by aminoacyl-tRNA synthetases.** *Nucleic acids research* 1981, **9:**3105-3117.

8. Chechetkin V: **Genetic code from tRNA point of view.** *Journal of theoretical biology* 2006, **242:**922-934.

9. Brown JR, Doolittle WF: **Root of the universal tree of life based on ancient aminoacyl-tRNA synthetase gene duplications.** *Proceedings of the National Academy of Sciences* 1995, **92:**2441-2445.

10. Pfeifer M, Kugler KG, Sandve SR, Zhan B, Rudi H, Hvidsten TR, Mayer KF, Olsen O-A, Consortium IWGS: **Genome interplay in the grain transcriptome of hexaploid bread wheat.** *Science* 2014, **345:**1250091.

11. Agris PF, Vendeix FA, Graham WD: **tRNA’s wobble decoding of the genome: 40 years of modification.** *Journal of molecular biology* 2007, **366:**1-13.

12. Zrenner R, Stitt M, Sonnewald U, Boldt R: **Pyrimidine and purine biosynthesis and degradation in plants.** *Annu Rev Plant Biol* 2006, **57:**805-836.

13. Azevedo R, Lancien M, Lea P: **The aspartic acid metabolic pathway, an exciting and essential pathway in plants.** *Amino acids* 2006, **30:**143-162.

14. Laplante M, Sabatini DM: **mTOR signaling in growth control and disease.** *Cell* 2012, **149:**274-293.

15. Asnaghi L, Bruno P, Priulla M, Nicolin A: **mTOR: a protein kinase switching between life and death.** *Pharmacological Research* 2004, **50:**545-549.

16. Herrmann KM: **The shikimate pathway: early steps in the biosynthesis of aromatic compounds.** *The Plant Cell* 1995, **7:**907-919.

17. Dudareva N: **Aromatic Amino Acid Network: Biosynthesis, Regulation and Transport.** *The FASEB Journal* 2015, **29:**103.102.

18. Kirma M, Araújo WL, Fernie AR, Galili G: **The multifaceted role of aspartate-family amino acids in plant metabolism.** *Journal of experimental botany* 2012**:**ers119.

19. Hildebrandt TM, Nesi AN, Araújo WL, Braun H-P: **Amino acid catabolism in plants.** *Molecular plant* 2015, **8:**1563-1579.

20. Jander G, Joshi V: **Aspartate-derived amino acid biosynthesis in Arabidopsis thaliana.** *The Arabidopsis book/American Society of Plant Biologists* 2009, **7:**e0121.

21. Locasale JW: **Serine, glycine and one-carbon units: cancer metabolism in full circle.** *Nature Reviews Cancer* 2013, **13:**572-583.

22. Foyer CH, Halliwell B: **The presence of glutathione and glutathione reductase in chloroplasts: a proposed role in ascorbic acid metabolism.** *Planta* 1976, **133:**21-25.

23. Rennenberg H, Brunold C: **Significance of glutathione metabolism in plants under stress.** In *Progress in botany.* Springer; 1994: 142-156

24. Forde BG, Lea PJ: **Glutamate in plants: metabolism, regulation, and signalling.** *Journal of Experimental Botany* 2007, **58:**2339-2358.

25. Michaeli S, Fromm H: **Closing the Loop on the GABA Shunt in Plants: Are GABA metabolism and signaling entwined?** *Frontiers in Plant Science* 2015, **6:**419.

26. Blumenthal S, Hendrickson H, Abrol Y, Conn EE: **Cyanide metabolism in higher plants III. The biosynthesis of β-cyanoalanine.** *Journal of Biological Chemistry* 1968, **243:**5302-5307.

27. Szabados L, Savouré A: **Proline: a multifunctional amino acid.** *Trends in plant science* 2010, **15:**89-97.

28. Slocum RD: **Genes, enzymes and regulation of arginine biosynthesis in plants.** *Plant Physiology and Biochemistry* 2005, **43:**729-745.

29. Kishor PK, Sangam S, Amrutha R, Laxmi PS, Naidu K, Rao K, Rao S, Reddy K, Theriappan P, Sreenivasulu N: **Regulation of proline biosynthesis, degradation, uptake and transport in higher plants: its implications in plant growth and abiotic stress tolerance.** *Curr Sci* 2005, **88:**424-438.

30. Delauney AJ, Verma DPS: **Proline biosynthesis and osmoregulation in plants.** *The plant journal* 1993, **4:**215-223.

31. Rangan P, Subramani R, Kumar R, Singh AK, Singh R: **Recent advances in polyamine metabolism and abiotic stress tolerance.** *BioMed research international* 2014, **2014:**239621.

32. Pilon-Smits EA, Quinn CF: **Selenium metabolism in plants.** In *Cell biology of metals and nutrients.* Springer; 2010: 225-241

33. Yao X, Chu J, Wang G: **Effects of selenium on wheat seedlings under drought stress.** *Biological trace element research* 2009, **130:**283-290.

34. Whanger P: **Selenocompounds in plants and animals and their biological significance.** *Journal of the American College of Nutrition* 2002, **21:**223-232.

35. Nelson DL, Cox MM: *Lehninger principles of biochemistry.* 5th edn: Freeman; 2013.

36. Schnarrenberger C: **Characterization and compartmentation, in green leaves, of hexokinases with different specificities for glucose, fructose, and mannose and for nucleoside triphosphates.** *Planta* 1990, **181:**249-255.

37. Holden HM, Rayment I, Thoden JB: **Structure and function of enzymes of the Leloir pathway for galactose metabolism.** *Journal of Biological Chemistry* 2003, **278:**43885-43888.

38. Roberts R: **The Formation of Uridine Diphosphate-Glucuronic Acid in Plants.** *Journal of Biological Chemistry* 1971, **246:**4995-5002.

39. Peregrín-Alvarez JM, Sanford C, Parkinson J: **The conservation and evolutionary modularity of metabolism.** *Genome biol* 2009, **10:**R63.

40. Loewus F: **Carbohydrate interconversions.** *Annual Review of Plant Physiology* 1971, **22:**337-364.

41. Higgins JP, Tuttle TD, Higgins CL: **Energy beverages: content and safety.** In *Mayo Clinic Proceedings*. Elsevier; 2010: 1033-1041.

42. Valpuesta V, Botella MA: **Biosynthesis of L-ascorbic acid in plants: new pathways for an old antioxidant.** *Trends in plant science* 2004, **9:**573-577.

43. Fernie AR, Carrari F, Sweetlove LJ: **Respiratory metabolism: glycolysis, the TCA cycle and mitochondrial electron transport.** *Current opinion in plant biology* 2004, **7:**254-261.

44. Kruger NJ, von Schaewen A: **The oxidative pentose phosphate pathway: structure and organisation.** *Current opinion in plant biology* 2003, **6:**236-246.

45. Hatefi Y: **The mitochondrial electron transport and oxidative phosphorylation system.** *Annual review of biochemistry* 1985, **54:**1015-1069.

46. Moore A, Rich P: **Organization of the respiratory chain and oxidative phosphorylation.** In *Higher Plant Cell Respiration.* Springer; 1985: 134-172

47. Noctor G, Hager J, Li S: **Biosynthesis of NAD and its manipulation in plants.** *Advances in botanical research* 2011, **58:**153-201.

48. Douce R, Bourguignon J, Neuburger M, Rébeillé F: **The glycine decarboxylase system: a fascinating complex.** *Trends in plant science* 2001, **6:**167-176.

49. Morrison I: **The structure of the chlorophyll-containing cross cells and tube cells of the inner pericarp of wheat during grain development.** *Botanical Gazette* 1976**:**85-93.

50. Wirth E, Kelly G, Fischbeck G, Latzko E: **Enzyme activities and products of CO 2 fixation in various photosynthetic organs of wheat and oat.** *Zeitschrift für Pflanzenphysiologie* 1977, **82:**78-87.

51. Wang P, Gao J, Wan C, Zhang F, Xu Z, Huang X, Sun X, Deng X: **Divinyl chlorophyll (ide) a can be converted to monovinyl chlorophyll (ide) a by a divinyl reductase in rice.** *Plant physiology* 2010, **153:**994-1003.

52. Rebeiz CA: *Chlorophyll Biosynthesis and Technological Applications.* Springer; 2014.

53. Singal H, Sheoran I, Singh R: **In vitro enzyme activities and products of 14CO2 assimilation in flag leaf and ear parts of wheat (Triticum aestivum L.).** *Photosynthesis research* 1986, **8:**113-122.

54. Rangan P, Furtado A, Henry RJ: **New evience for grain specific C4 photosynthesis in wheat.** *Scientific reports* 2016.

55. Parimalan R, Agnelo F, Robert H: **C4 photosynthesis in wheat seed?** *Frontiers in Plant Science* 2016, **7:**1537.

56. Schmid MK, Ohlrogge BJ: **Lipid metabolism in plants.** In *Biochemistry of lipids, lipoproteins and membranes.* 4 edition. Edited by Vance D, Vance J: Elsevier Science; 2002: 93-126

57. Fahy E, Subramaniam S, Brown HA, Glass CK, Merrill AH, Murphy RC, Raetz CR, Russell DW, Seyama Y, Shaw W, et al: **A comprehensive classification system for lipids.** *Journal of lipid research* 2005, **46:**839-862.

58. George EF: *Plant propagation by tissue culture. Part 1: the technology.* 2

edn: Exegetics limited; 1993.

59. Bishop GJ, Koncz C: **Brassinosteroids and plant steroid hormone signaling.** *The Plant Cell* 2002, **14:**S97-S110.

60. Khripach V, Zhabinskii V, de Groot A: **Twenty years of brassinosteroids: steroidal plant hormones warrant better crops for the XXI century.** *Annals of Botany* 2000, **86:**441-447.

61. Asensi-Fabado MA, Munné-Bosch S: **Vitamins in plants: occurrence, biosynthesis and antioxidant function.** *Trends in plant science* 2010, **15:**582-592.

62. Dong H, Beer S: **Riboflavin induces disease resistance in plants by activating a novel signal transduction pathway.** *Phytopathology* 2000, **90:**801-811.

63. Deng B, Jin X, Yang Y, Lin Z, Zhang Y: **The regulatory role of riboflavin in the drought tolerance of tobacco plants depends on ROS production.** *Plant growth regulation* 2014, **72:**269-277.

64. Jabrin S, Ravanel S, Gambonnet B, Douce R, Rébeillé F: **One-carbon metabolism in plants. Regulation of tetrahydrofolate synthesis during germination and seedling development.** *Plant physiology* 2003, **131:**1431-1439.

65. Lähdesmäki P: **Biosynthesis of taurine peptides in brain cytoplasmic fraction in vitro.** *International journal of neuroscience* 1987, **37:**79-84.

66. Birdsall TC: **Therapeutic applications of taurine.** *Alternative medicine review: a journal of clinical therapeutic* 1998, **3:**128-136.

67. Terrill JR, Grounds MD, Arthur PG: **Taurine deficiency, synthesis and transport in the mdx mouse model for Duchenne Muscular Dystrophy.** *The international journal of biochemistry & cell biology* 2015, **66:**141-148.

68. Chesney RW, Hedberg GE, Rogers QR, Dierenfeld ES, Hollis BE, Derocher A, Andersen M: **Does taurine deficiency cause metabolic bone disease and rickets in polar bear cubs raised in captivity?** In *Taurine 7.* Springer; 2009: 325-331

69. Aruoma IO, Halliwell B, Hoey MB, Butler J: **The antioxidant action of taurine, hypotaurine, and their metabolic precursors.** *Biochemical Journal* 1988, **256:**251-255.

70. Chae L, Kim T, Nilo-Poyanco R, Rhee SY: **Genomic signatures of specialized metabolism in plants.** *Science* 2014, **344:**510-513.

71. Pichersky E, Lewinsohn E: **Convergent evolution in plant specialized metabolism.** *Annual review of plant biology* 2011, **62:**549-566.

72. Vogt T: **Phenylpropanoid biosynthesis.** *Molecular plant* 2010, **3:**2-20.

73. Liu J, Osbourn A, Ma P: **MYB transcription factors as regulators of phenylpropanoid metabolism in plants.** *Molecular plant* 2015, **8:**689-708.

74. Xu W, Dubos C, Lepiniec L: **Transcriptional control of flavonoid biosynthesis by MYB–bHLH–WDR complexes.** *Trends in plant science* 2015, **20:**176-185.

75. Winkel-Shirley B: **Flavonoid biosynthesis. A colorful model for genetics, biochemistry, cell biology, and biotechnology.** *Plant physiology* 2001, **126:**485-493.

76. Winkel-Shirley B: **Biosynthesis of flavonoids and effects of stress.** *Current opinion in plant biology* 2002, **5:**218-223.

77. Himi E, Noda K: **Red grain colour gene (R) of wheat is a Myb-type transcription factor.** *Euphytica* 2005, **143:**239-242.

78. Zeven A: **Wheats with purple and blue grains: a review.** *Euphytica* 1991, **56:**243-258.

79. Knievel D, Abdel-Aal E-S, Rabalski I, Nakamura T, Hucl P: **Grain color development and the inheritance of high anthocyanin blue aleurone and purple pericarp in spring wheat (Triticum aestivum L.).** *Journal of Cereal Science* 2009, **50:**113-120.

80. Lichtenthaler HK: **The 1-deoxy-D-xylulose-5-phosphate pathway of isoprenoid biosynthesis in plants.** *Annual review of plant biology* 1999, **50:**47-65.

81. Boucher Y, Doolittle WF: **The role of lateral gene transfer in the evolution of isoprenoid biosynthesis pathways.** *Molecular microbiology* 2000, **37:**703-716.

82. Basset G: **A Systems Biology Approach to Detect the Hidden Reactions of Terpenoid Quinone Metabolism.** *The FASEB Journal* 2015, **29:**103.103.

83. He X, Zhang Y, He Z, Wu Y, Xiao Y, Ma C, Xia X: **Characterization of phytoene synthase 1 gene (Psy1) located on common wheat chromosome 7A and development of a functional marker.** *Theoretical and Applied Genetics* 2008, **116:**213-221.

84. Cenci A, Somma S, Chantret N, Dubcovsky J, Blanco A: **PCR identification of durum wheat BAC clones containing genes coding for carotenoid biosynthesis enzymes and their chromosome localization.** *Genome* 2004, **47:**911-917.

85. Cazzonelli CI, Pogson BJ: **Source to sink: regulation of carotenoid biosynthesis in plants.** *Trends in plant science* 2010, **15:**266-274.

86. Wu Y, Zhou K, Toyomasu T, Sugawara C, Oku M, Abe S, Usui M, Mitsuhashi W, Chono M, Chandler PM, Peters RJ: **Functional characterization of wheat copalyl diphosphate synthases sheds light on the early evolution of labdane-related diterpenoid metabolism in the cereals.** *Phytochemistry* 2012, **84:**40-46.

87. Zhou K, Xu M, Tiernan M, Xie Q, Toyomasu T, Sugawara C, Oku M, Usui M, Mitsuhashi W, Chono M, et al: **Functional characterization of wheat ent-kaurene (-like) synthases indicates continuing evolution of labdane-related diterpenoid metabolism in the cereals.** *Phytochemistry* 2012, **84:**47-55.

88. Olszewski N, Sun T-p, Gubler F: **Gibberellin signaling biosynthesis, catabolism, and response pathways.** *The Plant Cell* 2002, **14:**S61-S80.

89. Frey M, Schullehner K, Dick R, Fiesselmann A, Gierl A: **Benzoxazinoid biosynthesis, a model for evolution of secondary metabolic pathways in plants.** *Phytochemistry* 2009, **70:**1645-1651.
